# No developmental fronto-parietal shift in brain activation during mental arithmetic across the lifespan: A registered report

**DOI:** 10.1371/journal.pone.0353139

**Published:** 2026-08-05

**Authors:** Christina Artemenko, Mine Avcil

**Affiliations:** 1 Department of Psychology, University of Tuebingen, Tuebingen, Germany; 2 LEAD Graduate School & Research Network, University of Tuebingen, Tuebingen, Germany; 3 German Center for Mental Health (DZPG), Tuebingen, Germany; RIKEN CBS: RIKEN Noshinkei Kagaku Kenkyu Center, JAPAN

## Abstract

Arithmetic processing is represented in a fronto-parietal network of the brain. However, activation within this network is thought to undergo a developmental shift from domain-general cognitive processing in the frontal cortex towards domain-specific magnitude processing in the parietal cortex. This assumption is primarily based on findings in children and young adults. In this registered report, we set out to replicate the proposed gradual fronto-parietal activation shift in arithmetic processing and, for the first time, to explore how neural development of arithmetic continues during aging. This registered report focuses on the behavioral and neural correlates of arithmetic and arithmetic complexity across the lifespan, i.e., childhood, when arithmetic is first learned; young adulthood, when arithmetic skills are well established already, and old age, when individuals have lifelong arithmetic experience. Therefore, brain activation during mental arithmetic was measured in children, younger adults, and older adults using functional near-infrared spectroscopy (fNIRS). Arithmetic complexity was manipulated via carry and borrow operations in two-digit addition and subtraction. The results provide evidence that increasing arithmetic complexity is associated with increased activation in the fronto-parietal network across all age groups. Even though behavioral carry and borrow effects decrease during development, suggesting improvements in place-value processing, the underlying categorical and continuous processing characteristics and cortical activation remain relatively stable. While frontal activation may decrease from childhood to adulthood, parietal activation does not show a corresponding increase. Thus, the developmental fronto-parietal shift in arithmetic processing might only apply to single-digit arithmetic – but not to two-digit arithmetic across the lifespan. Overall, these results suggest that arithmetic skills acquired in childhood are maintained throughout the lifespan.

## Introduction

Arithmetic skills are acquired in school and are later important for everyday life. Hence, it is essential to better understand the underlying mechanisms of these skills not only in children, who have just learnt these skills, and in adults, whose arithmetic skills are well established, but also in older adults, because deficits in these skills have a detrimental impact on independent living. Therefore, the current study sets out to investigate the behavioral and neural correlates of arithmetic across the lifespan.

Arithmetic is represented in a fronto-parietal network in the brain [1], for meta-analyses in children and adults see [2], for a review in children see [3], for a model and its extensions in adults see [4, 5], but the extent of frontal and parietal activation in this network changes during development. From childhood to adulthood, frontal activation in the inferior frontal gyrus (IFG) and middle frontal gyrus (MFG) decreases, while parietal activation in the intraparietal sulcus (IPS), supramarginal gyrus (SMG), and angular gyrus (AG) increases [6–10]. The brain areas in this fronto-parietal network fulfill different functions during mental arithmetic [4,5]: Activation of the IFG and MFG is mainly associated with domain-general processes, such as working memory required for arithmetic; however, the exact location in the prefrontal cortex depends on the specific task demands [11]. Activation of the IPS, located at the border between the superior parietal lobule (SPL) and the inferior parietal lobule (IPL), reflects domain-specific number magnitude processing, and thus, an age-related activation increase indicates functional specialization [7]. Less deactivation of the SMG and AG (constituting the IPL) is specific for arithmetic fact retrieval, i.e., the automatic mapping of single-digit arithmetic problems to their solutions [12]. Altogether, there is evidence for a gradual developmental shift from domain-general frontal activation towards more specific parietal activation within the fronto-parietal network of arithmetic [6,13] when comparing children and adults.

Moreover, the question arises as to how these developmental changes progress during aging. With increasing age, general cognitive capacities decline [14,15]. Cognitive processing in older adults is especially limited by decreased working memory capacity and processing speed. Although some numerical abilities are affected by age-related changes, arithmetic skills are known to be mainly preserved in older adults [16, 17, for a review see 18]. For instance, arithmetic fact knowledge and automated counting procedures in the single-digit range were shown to be equal or even superior to younger adults [18,19]. Since neuroimaging research on arithmetic in older adults has never been conducted so far, this project on the behavioral and neural correlates of arithmetic during development across the whole lifespan opens a new research field in numerical cognition. The investigation of the mechanisms underlying arithmetic in the elderly brain will provide insights into whether the gradual developmental fronto-parietal shift continues during aging, and whether arithmetic is preserved during aging or shows deficits due to the general cognitive decline, thus requiring compensation.

When studying the underlying domain-general and domain-specific processes of arithmetic across the lifespan, the complexity of the arithmetic task plays an important role. While the majority of neuroimaging studies on arithmetic mainly focus on single-digit arithmetic, after their first year of school, children mainly calculate with multi-digit numbers, and adults need multi-digit arithmetic in their daily lives well into old age, e.g., when handling finances, comparing prices while shopping, managing their time, or calculating weights while cooking. Knowledge of the place-value system is crucial for multi-digit number processing and consists of three levels: place identification, place-value activation, and place-value computation [20]. Multi-digit arithmetic is especially difficult when it requires operations across place-values, i.e., place-value computation [20]. This is the case for the carry operation in addition (when the sum of the units exceeds 9 so that the decade digit of the unit sum needs to be carried to the decade sum; e.g., 58 + 36 vs. 51 + 43) and the borrow operation in subtraction (when the minuend unit is smaller than the subtrahend unit so that a decade digit of the minuend needs to be borrowed for the difference of the units; e.g., 94–36 vs. 94–43).

The carry and borrow operations increase the difficulty of addition and subtraction problems as reflected by behavioral [21,22] and neural effects [23–26]. The behavioral carry and borrow effects were already demonstrated in children [27–29], in adolescents [8,30], in younger adults [21,22,31], and in older adults [32,33]. The difficulty, as indicated by increases in reaction time and error rate, is attributed to higher working memory load, reflecting domain-general processes when comparing problems with carry and borrow operations to problems without them, i.e., the categorical carry and borrow effects [22,34,35]. While the categorical carry and borrow operations require place-value computation – the highest level of place-value processing – the continuous carry and borrow operations instead require place-value activation [20]. Continuous processing characteristics of the carry operation are indicated by the unit sum (e.g., 58 + 36 has a unit sum of 8 + 6 = 14), which theoretically ranges from 0 to 18 (carry operation necessary when unit sum ≥ 10); continuous processing characteristics of the borrow operation are indicated by the unit difference (e.g., 94–36 has a unit difference of 4–6 = –2), which theoretically ranges from –9 to +9 (borrow operation necessary when unit difference < 0). The carry operation is characterized by both categorical and continuous processing characteristics [31,36]. The nature of the borrow operation, however, remains unclear, since continuous processing characteristics might not necessarily increase the difficulty of subtraction in a similar way as it is the case for addition [37].

During development, children get better at arithmetic and place-value processing [38, for a review see 39]. Children learn the carry and borrow operations for single-digit and two-digit arithmetic in the first two years of elementary school and afterwards are able to use these skills [27]. Place identification, the first level of place-value processing, serves as a precursor for arithmetic performance and place-value computation later in elementary school [29]. The carry and borrow effects decrease during adolescence from grade 5–7 and to young adulthood [30], but not before [8,27,40]. Besides general increases in reaction times and error rates during aging, the carry and borrow operations are not impaired and might be even superior in older as compared to younger adults, as reflected by similar or smaller carry and borrow effects [32,33,41]. This reflects a general decrease in the carry and borrow effects during lifespan development with increasing proficiency in place-value processing. Furthermore, the underlying processing characteristics of these effects might change, since the carry and borrow effects seem to be categorical effects in elementary school children [27], while the carry effect in younger adults relies on continuous processing characteristics as well, as assessed by the unit sum [26,31,36]. This suggests that primarily domain-general processes are driving the carry and borrow effects in children, while in younger adults, domain-specific processes are additionally driving the carry effect. Considering the general cognitive decline during aging, the carry and borrow effects might be mainly driven by domain-specific processes in older adults, but empirical evidence is still missing. The next step is now to replicate the developmental changes in arithmetic in general and in place-value computation in particular (i.e., carry and borrow effects), to identify the underlying processing characteristics (i.e., categorical and continuous aspects) across the lifespan, and to complement the behavioral findings by neural data.

The neural representations of the carry and borrow effects are located in the fronto-parietal network of arithmetic processing [26]. Mainly, carry and borrow effects are associated with higher prefrontal activation in the left IFG and bilateral MFG [23–26,42,43], mostly reflecting domain-general demands like working memory for task difficulty due to the categorical effects [44,45]. Additionally, parietal activation, particularly in the left IPS, was observed with increasing unit sum or when carry and borrow effects were confounded with problem size, mostly reflecting domain-specific magnitude processing associated with the continuous effects [23,24,26]. Furthermore, the only study on the neural correlates of the carry and borrow effects that was not conducted in younger adults found the left AG to be reversely related to the carry effect in adolescents, reflecting the role of arithmetic fact retrieval for decomposed addition [8]. Taken together, the carry and borrow effects are associated with increases in frontal and parietal activation. Due to the developmental fronto-parietal shift in brain activation for arithmetic in general, the neural activation might also change for processing arithmetic complexity. For instance, frontal activation associated with the carry and borrow operations might decrease during lifespan development due to automatization, since the use of domain-general processes becomes more efficient from childhood to adulthood, and the general cognitive decline restricts further efficient use of domain-general processes during aging. On the other hand, a study on interindividual differences in math ability showed that smaller behavioral carry and borrow effects might be associated with larger neural effects (in high- as compared to low-skilled individuals), since high-skilled individuals efficiently used the frontal resources for the carry and borrow operations whereas low-skilled individuals needed these resources even for problems without carry or borrow operation [25]. Thus, a decrease in the behavioral carry and borrow effects during lifespan development – as individuals become more proficient in the carry and borrow operations – might be associated with an increase in the neural carry and borrow effects in frontal brain regions. The current study aims to investigate the behavioral and neural correlates of the carry and borrow effects in children, younger adults, and older adults, and to explore developmental changes.

Arithmetic processing in general and the carry and borrow effects in particular are represented in a fronto-parietal network of arithmetic processing. Here, we address the question of how arithmetic and the underlying brain activation in the arithmetic network change during development across the lifespan. The study targets crucial stages of development: elementary school children in grades 3 and 4, because they just acquired the skills for two-digit arithmetic and thus serve as a starting point of lifespan development, younger adults, because they have already established their arithmetic skills and thus serve as a reference for development, and older adults, because they are experienced in using their arithmetic skills but might show a general cognitive decline and thus serve as a final point of lifespan development. A gradual neural activation shift is hypothesized from frontal activation, mostly representing domain-general processes, to parietal activation, mostly representing domain-specific numerical processes, during development. Using three different approaches [29], we address the following hypotheses at behavioral and neural levels:

- H1: In a task-based approach, arithmetic performance is expected to be better in younger adults than in children and in older adults. According to the developmental fronto-parietal shift for arithmetic processing, children should show increased frontal activation (left IFG and bilateral MFG) and less parietal activation (left IPS) in comparison to younger adults, replicating previous research. Moreover, the neural correlates of arithmetic will be explored in older adults: if the developmental fronto-parietal shift generalizes to the whole lifespan, older adults might show less frontal activation (left IFG and bilateral MFG) and increased parietal activation (left IPS) in comparison to younger adults; however, if arithmetic is not affected by aging, activation might be similar in older and younger adults; finally, if arithmetic is affected by aging and needs compensation, older adults might show increased frontal activation in comparison to younger adults.
- H2: In an effect-based approach, the carry and borrow effects are expected to decrease arithmetic performance at the behavioral level (i.e., reaction times and error rates) and to be associated with larger frontal activation (left IFG and bilateral MFG) at the neural level in all age groups. Regarding the lifespan development, the behavioral carry and borrow effects are expected to be larger in children than in younger adults and larger or similar in young adults as compared to older adults, replicating previous research. The neural development of the effects will be explored here for the first time: the neural carry and borrow effects might either decrease during the lifespan, reflecting the fronto-parietal activation shift particularly for complex arithmetic, or increase during the lifespan, reflecting the more efficient use of frontal resources with increasing performance.
- H3: In an effect-based approach on the underlying processing characteristic, the carry and borrow effects are expected to be rather categorical in children, both categorical and continuous in younger adults, and rather continuous in older adults. At the neural level, the continuous carry and borrow effects (unit sum and unit difference) should rely on parietal activation (left IPS), while the categorical carry and borrow effects mainly rely on frontal activation (left IFG and bilateral MFG) across age groups.

The carry and borrow effects will be investigated concerning arithmetic complexity in two-digit addition and subtraction, respectively. The processes underlying these arithmetic operations are similar, while subtraction is more difficult than addition [25]. However, there is no evidence yet for operation-specific differences in relation to lifespan development. To assess brain activation during two-digit arithmetic, the optical neuroimaging method fNIRS will be used. Compared to fMRI, fNIRS is less restrictive, allows for an upright body position, and is relatively insensitive to motion artifacts – albeit at the cost of lower spatial and depth resolution [46]. The current study makes use of the advantages of fNIRS to study arithmetic in an ecologically valid task paradigm (verbal production) in critical populations such as children and older adults.

## Methods

### Participants

Three age groups were considered: children (3^rd^ and 4^th^ grade), younger adults (18–34 years), and older adults (above 60 years). In the present study, *N* = 60 children (25 female, 34 male, 1 missing value; age: *M* ± *SD* = 9.46 ± 0.61, *range* = 8.33–10.67 years) with *M* ± *SD* = 3.47 ± 0.50 years of education, *N* = 62 younger adults (52 female, 10 male; age: *M* ± *SD* = 22.40 ± 2.92, *range* = 18.25–32.00 years) with *M* ± *SD* = 13.55 ± 3.81 years of education, and *N* = 61 older adults (28 female, 33 male; age: *M* ± *SD* = 68.56 ± 5.41, *range* = 60.08–83.92 years) with *M* ± *SD* = 18.51 ± 5.56 years of education participated. All subjects were right-handed, native German speakers (or at least school education in German), with no history of neurological or mental disorders, and without a disease that influences brain metabolism. Informed consent was obtained via mouse click from all adult participants, from the parents of the participating children, and from the participating children in an age-appropriate, simplified form. For participation, all subjects received monetary compensation and children additionally a small gift. The study was approved by the Ethics Committee for Psychological Research of the University of Tuebingen and data collection took place from 10/2021–01/2025.

To emphasize the focus on healthy aging in the current study, older adults were additionally assessed by the Montreal Cognitive Assessment [MoCA; 47], a brief cognitive screening tool for mild cognitive impairment. This instrument measures cognitive abilities such as short-term memory, visuo-spatial and executive functions, attention, language, and orientation to time and space. A cut-off score of ≥ 26 (*theoretical range* = 0–30 with correction in case of ≤ 12 years of education) was used to exclude cognitive impairment in older adults (*n* = 5). Additionally, processing speed, working memory, and verbal and non-verbal intelligence served as control measures to compare general cognitive abilities between the age groups.

### Arithmetic task

The arithmetic task consisted of two-digit addition and subtraction problems with two operands resulting in a two-digit solution [https://osf.io/6emdy/]. The carry and borrow operations were manipulated categorically, i.e., addition problems with and without carrying (e.g., 51 + 43 vs. 58 + 36) as well as subtraction problems with and without borrowing (e.g., 94–43 vs. 94–36), and continuously, i.e., unit sum in addition (e.g., 14 in 58 + 36) and unit difference in subtraction (e.g., – 2 in 94–36).

The stimulus set consisted of 128 arithmetic problems with 32 trials per condition, i.e., addition with/without carrying and subtraction with/without borrowing. The stimulus generation considered unit sum in addition and unit difference in subtraction to be relatively equally distributed. This means that addition problems (*a* + *b* = *r*) could not be directly transformed into subtraction problems by inversion (*r* – *b* = *a*). Nevertheless, the numerical properties were matched across conditions: *a* and *b* were closely matched in their numerical magnitude and parity; the position of the larger operand was counterbalanced; pure decades as well as ties within and between *a*, *b*, and *r* were excluded [25, 42, for decades see 48, for ties see 49].

The task was computerized in the program OpenSesame [50]. Each arithmetic problem was presented centered in white against a black background. In an oral production paradigm, the subjects were asked to mentally solve the problem as quickly and accurately as possible and to respond while pressing the space bar. Button press and button release were recorded and the experimenter blindly noted the given responses. Each stimulus was presented with a time limit of 30 s and disappeared upon button press to emphasize mental arithmetic before responding. In the inter-trial interval, a black screen was shown for a duration of 4–7 s (jittered in steps of 0.5 s, mean of 5.5 s), including a white fixation point in the last 0.5 s. The 128 trials were presented in 4 runs of 32 trials each (8 trials per condition) and the trial order was pseudo-randomized for every subject with no more than two trials of the same condition presented consecutively. As dependent variables, error rates (ER) are defined as the number of incorrectly solved and time-out trials divided by the total number of completed trials, and reaction times (RT) as the duration between stimulus onset and button press.

### Cognitive tests

Processing speed was assessed by the subtest symbol search of the German version of the Wechsler Adult Intelligence Scale IV [WAIS-IV; 51]. In this paper-pencil test, subjects were asked to decide whether or not two target symbols were present among a group of five symbols. The overall time limit was 120 s for a maximum of 60 items. The raw score (*theoretical range* = 0–60) is defined as the number of correctly solved items minus the number of incorrectly solved items (unsolved items are not considered). The retest reliability of the German version is good (*r*_*tt*_ = .81).

Working memory was assessed by a verbal 2-back paradigm with letters [https://osf.io/6emdy/; 52]. The task was computerized in the program OpenSesame [50]. In this task, subjects were asked to determine for every letter (consonants in lower and upper case) whether it matched the letter presented two positions before or not by a button press. The 92 trials consisted of 30 match trials (match to the letter presented two positions before), 48 mismatch trials (no match to a letter presented one to five positions before), 6 1-back lures (match to the letter presented one position before), 6 3-back lures (match to the letter presented three positions before), and 2 start letters (presented first). Each stimulus was presented until the button press, with a time limit of 2.5 s, followed by an SOA of 3 s. Accuracy (ACC) is defined as the number of correct trials divided by the total number of trials, and RT as the duration between stimulus onset and button press. Note that the group comparisons focus on ACC.

Intelligence was assessed by the German version of the Reynolds Intellectual Screening Test [RIST; 53], consisting of subtests for verbal and nonverbal intelligence. Depending on age, each subtest starts at a certain item (with the option to return to preceding items until two consecutive items are correctly solved on the first attempt) and stopped when three consecutive items are not correctly solved. In the subtest “guess what”, indicating verbal intelligence, subjects were orally asked to find out the concept that matches the given two to four clues. With a maximum of 62 orally presented items, the raw score (*theoretical range* = 0–62) is defined as the sum of all correctly solved items (including the unsolved items before the age-dependent start item). In the subtest “odd item out”, indicating nonverbal intelligence, subjects were asked to choose the picture that does not belong to a set of five to seven pictures. The time limit was 30 s for the first attempt and 20 s for the second attempt (if incorrectly or unsolved in the first attempt), with a maximum of 51 visually presented items. The raw score (*theoretical range* = 0–102) is calculated as the double sum of all correctly solved items in the first attempt (including the unsolved items before the age-dependent start item) and the single sum of all correctly solved items in the second attempt. Raw scores from both subtests were further transformed into *T* scores (*M* = 50, *SD* = 10) based on German age norms and subsequently converted into IQ scores (*M* = 100, *SD* = 15). The reliability of the German version is good (Cronbach’s *α* of.92 for verbal intelligence and.90 for nonverbal intelligence).

### Procedure

During the fNIRS measurements, the arithmetic task was conducted in a light-attenuated room. Afterwards, processing speed, working memory, and intelligence were assessed. Each test was preceded by instructions and practice items. The practice phase of the arithmetic task consisted of 12 trials to familiarize the subjects with the response format (which could be repeated). Finally, a screening of cognitive abilities was conducted for older adults only.

### fNIRS data acquisition

**Fig 1:**
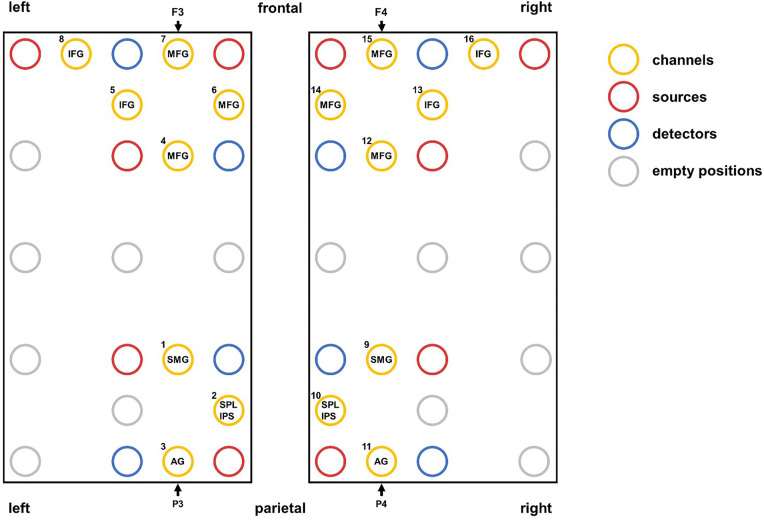
fNIRS probesets covering frontal and parietal areas of the left and right hemispheres. The probesets were fixed at P3/P4 and oriented towards F3/F4; the positions of channels and optodes (sources and detectors) including empty positions are marked [cf. 25]. *Abbreviations of the channel labels:* IFG – inferior frontal gyrus, MFG – middle frontal gyrus, SPL/IPS – superior parietal lobule/intraparietal sulcus, SMG – supramarginal gyrus, AG – angular gyrus.

The fNIRS data was acquired using the continuous wave ETG-4000 Optical Topography System (Hitachi Medical Corporation, Tokyo, Japan). This fNIRS device uses wavelengths of 695 ± 20 nm and 830 ± 20 nm as light sources and a sampling rate of 10 Hz. The optodes (10 sources and 8 detectors) were embedded in a cap (Brain Products GmbH, Herrsching, Germany) with an inter-optode distance of 30 mm. The probesets consisted of 4 parietal channels (IPS, SMG, AG) and 5 frontal channels (MFG, IFG) per hemisphere (see Fig 1), which is a subset of channels that were previously used as a probeset [for more details on the location about the probeset see 25]. The correspondence of fNIRS channels to the underlying cortical areas was estimated based on a virtual registration method [54–56] and labeled according to the automated anatomic labeling (AAL) atlas [57].

### Data analysis

*Data exclusion for subjects.* Subjects were excluded from all analyses if the inclusion criteria were not met (regarding age, handedness, language, health, and, for older adults, cognition, *n* = 5), if more than 50% of the behavioral data of the arithmetic task was missing (due to dropout or experimental or technical problems, *n* = 6), or if accuracy in the arithmetic task was below 50% (because of the exclusion of incorrectly solved trials, *n* = 2). Moreover, an outlier analysis was conducted specific to the respective age group to exclude subjects deviating more than 3 median absolute deviations from the group’s median in RT of the arithmetic task from all analyses [58] (*n* = 4). Subjects were excluded only from neural data analyses in case of more than 50% missing neural data of the arithmetic task (due to dropout, experimental or technical problems, trial exclusion, or artifact rejection, *n* = 1) or in case of more than 5 noisy channels (*n* = 7). Note that the preregistered criterion for noisy channels was increased from 3 to 5, as noisy channels were excluded rather than interpolated during data preprocessing. Furthermore, a case-wise exclusion of subjects from the respective analysis of demographic variables (age, gender, education) or control measures (processing speed, working memory, and verbal and non-verbal intelligence) was applied in cases of missing or incomplete data, or an ACC below 33% in the working memory task (for details on data exclusion see Table A in S1 File).

*Data exclusion for trials.* Trials were excluded from the RT analyses (arithmetic and working memory task) as well as from the fNIRS analyses (arithmetic task) if the trial was not correctly solved, if RT was below 200 ms (anticipations), if RT deviated more than 3 median absolute deviations from the subject’s median in the respective task, or if the duration between button press and button release deviated more than 3 median absolute deviations from the subject’s median in the arithmetic task [58] (for details on data exclusion see Table A in S1 File).

*fNIRS data preprocessing.* fNIRS data analysis was conducted using the NIRS Brain AnalyzIR toolbox [59] in Matlab (version 2020b). The fNIRS signal was preprocessed by using the temporal derivative distribution repair [TDDR; 60] to correct for high-amplitude motion artifacts. The relative concentration changes of oxygenated (O_2_Hb) and deoxygenated hemoglobin (HHb) were calculated for every fNIRS channel. A bandpass filter of 0.005–0.2 Hz was applied to the fNIRS signal by using an infinite impulse response Butterworth filter with an order of 5. To reduce low-amplitude motion artifacts, correlation-based signal improvement [CBSI; 61] was used, which is based on the negative correlation between O_2_Hb and HHb and is considered one of the best artifact correction methods [62]. Remaining noisy or flat channels were excluded from analysis (rather than interpolated by surrounding channels, as preregistered, because the toolbox used does not support interpolation), and incorrectly solved trials as well as trials containing uncorrectable artifacts were excluded.

To analyze the fNIRS data within a model-based approach, the peak latency of the hemodynamic response function was determined by the overall maximum across channels, subjects, and conditions [in the interval between 4 and 10 s rounded to half a second;,25]. Although this resulted in an early peak of 6 s, all subsequent analyses did not produce meaningful results (for a discussion see Table C in S1 File). Therefore, a peak latency of 9 s was chosen based on a prior study using the same task [63, see also 64]. In the model-based approach, a general linear model (GLM) was computed for each channel, subject, and condition according to the hemodynamic response function. For every region of interest (10 ROIs: IFG, MFG, IPS, AG, SMG on the left and right hemisphere), the channel with the highest resulting beta value based on the grand average across conditions and subjects was used for the statistical analysis of the neural data.

*Statistical data analysis.* This study applies Bayesian hypothesis testing and thus Bayes factors (*BF*) were calculated that determine how much more likely the observed data is under the alternative hypothesis (*H_1_*) as compared to the null hypothesis (*H_0_*) for *BF*_*10*_ (evidence for a difference when *BF*_*10*_ > 1) and vice versa for *BF*_*01*_ (evidence for no difference when *BF*_*01*_ > 1), whereby *BF*_*01*_ = 1/*BF*_*10*_ [65]. *BF*s can be interpreted to provide anecdotal evidence for 1–3, moderate evidence for 3–10, strong evidence for 10–30, and very strong evidence for 30–100, and extreme evidence above 100 in favor of one hypothesis [66,67].

The statistical analyses in terms of Bayesian *t*-tests, Bayesian ANOVAs and Bayesian linear regressions were performed with JASP (Jeffreys’s Amazing Statistics Program, JASP Team, 2016). The analysis prior was set to a Cauchy prior scale of 0.707 for Bayesian *t*-tests, reflecting that *H_0_* and *H_1_* are equally likely to occur, to the default Cauchy prior of *r* = 0.5 for the fixed effects in Bayesian ANOVAs, to the default Jeffreys–Zellner–Siow prior of *r* = 0.354 for regression coefficients, and to a uniform model prior in Bayesian linear regressions. Bayesian ANOVAs and linear regressions set out to compare each model to the null model and Bayesian model averaging compared the models with the respective effect to otherwise equivalent models without that effect (analysis suggested by Sebastiaan Mathôt).

Prior to the analyses of the arithmetic task, cognitive abilities were analyzed: children and older adults were compared to younger adults regarding processing speed (raw scores), working memory (ACC), and verbal and non-verbal intelligence (IQ scores) by two-sided Bayesian independent samples *t*-tests. Next, the arithmetic task was analyzed in 3 age (children, younger adults, older adults) × 2 operation (addition, subtraction) × 2 complexity (without, with carry/borrow) Bayesian repeated measures ANOVAs [analysis over subjects, averaged over trials]. For effects with *BF*_*10*_ or *BF*_*01*_ ≥ 6, post-hoc two-sided tests were conducted for the contrast children vs. younger adults and the contrast younger adults vs. older adults by means of Bayesian independent samples *t*-tests, or for contrasting different conditions by means of Bayesian paired *t*-tests. Finally, multi-model Bayesian regressions were conducted with the categorical predictor carry/borrow (without vs. with) and the continuous predictor unit sum/difference, separately for addition and subtraction and for each age group [analysis over trials, averaged over subjects].

All confirmatory analyses were conducted on the dependent variables RT, indicating behavioral performance, and beta values, indicating neural activation for each ROI separately (left/right IFG, MFG, IPS). Planned exploratory analyses were conducted on ACC and beta values for neural activation in the other ROIs (left/right AG, SMG). While the neural analyses over subjects were conducted on beta values derived from subject-level GLMs, the neural analyses over trials were conducted on mean amplitudes in the interval from 3 to 9 s, which were baseline-corrected using the 3 s preceding each trial. This deviation from the GLM-based approach was chosen because estimates from subject-level GLMs based on single trials would not be reliable. To confirm that amplitude-based analyses revealed activation patterns similar to those obtained from GLM beta values, the neural analyses over subjects were repeated for amplitudes (for results see Table E in S1 File).

### Bayes factor design analysis

For sample size estimation, a sequential Bayes factor design with maximal *n* was used [68,69]. In this design, data collection (1) starts with a minimum sample size of *n*_*min*_ = 20 per group, (2) continues until a *BF*_*10*_ or *BF*_*01*_ ≥ 6 is obtained for all effects of interest, or (3) stops when a maximum sample size of *n*_*max*_ = 60 per group is reached. Data collection was completed upon reaching the final sample size per group, in accordance with the stopping rule. The properties of the research design were estimated with Monte Carlo simulations according to Schönbrodt and Wagenmakers [68]: The minimum sample size was set for reducing false positive rates, and the maximum sample size was set to ensure feasibility, while 80% of studies with an infinite sequential sampling would stop earlier than *n*_*max*_. If sampling is terminated because of reaching *n*_*max*_, only with a probability of 5% will the study obtain misleading evidence. This design detects an expected medium effect size of *δ* = 0.5 with a probability of 61% before the *n*_*max*_ is reached. The chosen medium effect size accounts for the bias of small samples in the reported large effect sizes ($\eta_{p}^{\text{2}}$ ≈.5) for differences between children, younger adults, and older adults in the neural distance effect [70], for large effect sizes (*d* ≈ 1.2–1.6) see also for children vs. adults: [71], for younger adults vs. older adults: [72].

The effects of interest according to the hypotheses included the main effect of age (ANOVAs) for RT and activation in left IFG, bilateral MFG, and left IPS according to H1; the main effect of complexity and the interaction effect of complexity and age (ANOVAs) for RT and activation in left IFG and bilateral MFG according to H2; the categorical carry/borrow effect and the continuous effect of unit sum/difference in each age group (regressions) in RT and neural activation in left IPS according to H3.

## Results

### Cognitive results

The cognitive tests revealed age-related differences between the samples (see Table 1). Processing speed was slower in children and older adults than in younger adults. Working memory performance was also lower in children and older adults than in younger adults; however, the n-back task turned out to be inappropriate for children and, to some extent, also for older adults (see Table A in S1 File). Intelligence scores were higher in children (verbal and non-verbal) and in older adults (non-verbal) than in younger adults.

**Table 1:** Age-related differences in cognitive tests and arithmetic tasks.

variable	children			younger adults			older adults			children vs. younger adults	younger vs. older adults
	*N*	*M*	*SD*	*N*	*M*	*SD*	*N*	*M*	*SD*	*BF*_*10*_	*BF*_*10*_
processing speed	54	25.15	5.20	36	42.67	7.04	48	32.00	6.40	> 100	> 100
working memory	39	0.71	0.12	56	0.91	0.07	46	0.82	0.11	> 100	> 100
verbal intelligence	55	109.25	7.31	59	103.46	10.35	51	107.77	11.68	33.05	1.31
non-verbal intelligence	55	106.38	8.83	59	99.19	7.81	51	105.29	12.34	> 100	15.15
arithmetic ACC	55	0.88	0.08	59	0.91	0.05	52	0.92	0.06	1.41	0.33
arithmetic RT	55	8967	3200	59	3653	879	52	3397	1136	> 100	0.45

*Notes*. Dependent measures included processing speed scores, working memory accuracy, IQ scores for intelligence, and arithmetic accuracy (ACC) and reaction time (RT in ms).

### Behavioral results

**Fig 2:**
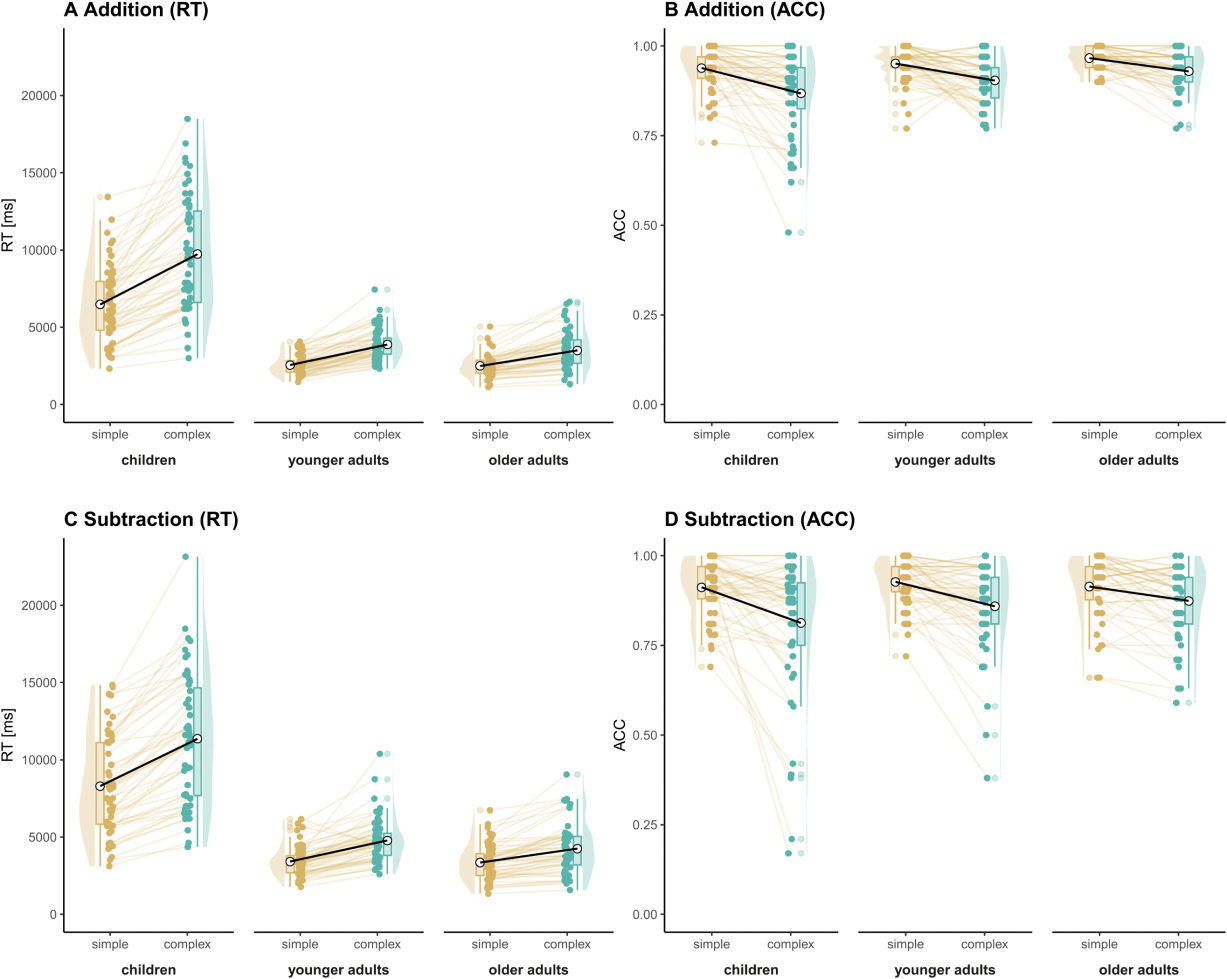
Behavioral results of the arithmetic task. Arithmetic performance is shown for reaction time (RT) and accuracy (ACC).

For the arithmetic task, the best-fitting model for RT included main effects of age group, operation, and complexity, as well as two-way interactions of age group with operation and complexity (*P*(*M*) = 0.053, *P*(*M*|data) = 0.865, *BF*_*M*_ > 100, *BF*_*10*_ > 100, error% = 9.999), with extreme evidence for all of these effects. The main effect of age group (*BF*_*incl*_ > 100) indicates that children (*M* = 8927 ms, *SD* = 157 ms) required more time to solve arithmetic problems than younger adults (*M* = 3672 ms, *SD* = 224 ms; *BF*_*10*_ > 100), while there was anecdotal evidence for no difference between younger and older adults (*M* = 3417 ms, *SD* = 226 ms; *BF*_*10*_ = 0.45, *BF*_*01*_ = 2.24). The main effect of operation (*BF*_*incl*_ > 100) indicates that solving subtraction problems took, on average, 1120 ms longer than solving addition problems. The main effect of complexity (*BF*_*incl*_ > 100) indicates that problems involving a carry or borrow operation needed, on average, 1814 ms longer to be solved than problems without a carry or borrow operation. The interaction between age group and operation indicates a larger operation effect in children (*BF*_*10*_ > 100), but no difference between younger and older adults (*BF*_*10*_ = 0.24, *BF*_*01*_ = 4.09). The interaction between age group and complexity indicates that the complexity effect was larger in children than in younger adults (*BF*_*10*_ > 100) and larger in younger adults than in older adults (*BF*_*10*_ = 7.75; see Fig 2). Planned exploratory analyses of ACC mainly revealed effects of operation and complexity (for details see S1 File).

Regression analyses of categorical (carry/borrow) and continuous predictors (unit sum/difference) revealed the following results for RT (for details see Table B in S1 File): For addition, the best-fitting models included both carry and unit sum in children, younger adults, and older adults, with extreme evidence for both predictors (except for only moderate evidence for carry in older adults). For subtraction, the best-fitting models included only borrow in children, younger adults, and older adults, with extreme evidence for this predictor. For children, the best-fitting model additionally included unit difference; however, the effect of unit difference was inconclusive (*BF*_*incl*_ = 1.45).

### fNIRS results

**Fig 3:**
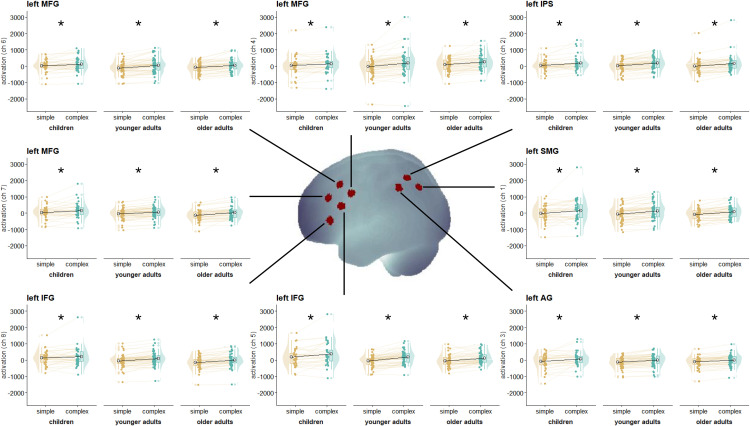
Neural effects of arithmetic complexity in the left brain hemisphere. Main effects of complexity with *BF*_*incl*_ > 3 are marked by *. *Abbreviations of brain regions:* IFG – inferior frontal gyrus, MFG – middle frontal gyrus, IPS – intraparietal sulcus, SMG – supramarginal gyrus, AG – angular gyrus.

**Fig 4:**
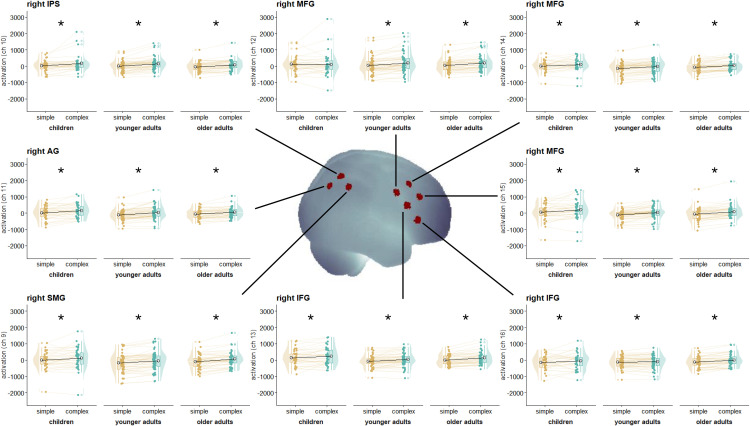
Neural effects of arithmetic complexity in the right brain hemisphere. Main effects of complexity with *BF*_*incl*_ > 3 are marked by *; the interaction of complexity with age in channel 12 is indicated by evidence for complexity effects in younger and older adults but not in children. *Abbreviations of brain regions:* IFG – inferior frontal gyrus, MFG – middle frontal gyrus, IPS – intraparietal sulcus, SMG – supramarginal gyrus, AG – angular gyrus.

Brain activation during arithmetic processing in the fronto-parietal network varied as a function of operation and complexity. All best-fitting models included main effects of operation and complexity, with extreme evidence. Activation in bilateral frontal (IFG and MFG) and parietal (IPS, AG, and SMG) brain regions was higher for subtraction than for addition, and for complex arithmetic involving carrying/borrowing compared to simple arithmetic without carrying/borrowing (see Table 2, Figs 3 and 4). Evidence for main effects of age group was largely inconclusive (see Table D in S1 File). Moreover, moderate evidence for an interaction between age group and complexity was found in the right MFG (*BF*_*incl*_ = 5.72; channel 12), indicating that the complexity effect was smaller in children than in younger adults (*BF*_*10*_ = 3.03; see Fig 4) and similar between younger and older adults (*BF*_*10*_ = 0.26; *BF*_*01*_ = 3.85). In all other brain regions, anecdotal to strong evidence was found against an interaction between age group and complexity (*BF*_*excl*_ > 2; see Table D in S1 File).

**Table 2:** Brain activation during arithmetic dependent on age, operation, and complexity.

brain area	hemisphere	ch	age	operation	complexity	age × operation	age × complexity	operation × complexity	age × operation × complexity
IFG	left	5	1.92	**> 100**	**> 100**	0.09	0.24	1.77	0.12
		8	2.26	**> 100**	**> 100**	0.18	0.17	0.53	0.10
	right	13	2.07	**> 100**	**> 100**	0.15	0.15	1.12	0.10
		16	0.25	**> 100**	**> 100**	0.41	0.40	0.36	0.10
MFG	left	4	0.46	**> 100**	**> 100**	0.06	0.43	1.25	0.12
		6	0.41	**> 100**	**> 100**	0.18	0.27	0.36	0.07
		7	0.66	**> 100**	**> 100**	0.68	0.11	0.15	0.19
	right	12	0.19	**> 100**	**> 100**	0.07	**5.72**	0.15	0.47
		14	0.57	**> 100**	**> 100**	0.33	0.10	0.12	0.39
		15	0.76	**> 100**	**> 100**	0.89	0.16	0.15	0.09
IPS	left	2	0.19	**> 100**	**> 100**	0.16	0.12	0.33	0.22
	right	10	0.68	**> 100**	**> 100**	0.10	0.03	0.14	0.03
AG	left	3	0.39	**> 100**	**> 100**	0.09	0.05	0.14	0.08
	right	11	0.61	**> 100**	**> 100**	0.08	0.11	0.13	0.19
SMG	left	1	0.38	**> 100**	**> 100**	0.03	0.23	0.18	0.10
	right	9	0.53	**> 100**	**> 100**	0.79	0.11	0.15	0.13

*Notes*. *BF*_*incl*_ are given for the analysis of effects in Bayesian ANOVAs (for further details see Table D in S1 File). *BF*_*incl*_ > 3 are marked in bold. Channels (ch) of ROIs are reported in the first line of each region. *Abbreviations of brain regions:* IFG – inferior frontal gyrus, MFG – middle frontal gyrus, IPS – intraparietal sulcus, SMG – supramarginal gyrus, AG – angular gyrus.

Exploratory results of amplitude and peak latency analyses are reported in S1 File (Tables E and F). The amplitude analyses provided convergent evidence for the GLM results, particularly for complexity effects across channels and the interaction between age group and complexity in the right MFG. However, the amplitude analysis also revealed age effects not captured by the GLM and less evidence for operation effects, possibly because the time course of the fNIRS signal and the underlying hemodynamic response varied depending on age, operation, complexity, and brain region, as further supported by the peak latency analysis.

Neural analyses of categorical (carry/borrow) and continuous predictors (unit sum/difference) yielded inconclusive results for all age groups, both operations, and all channels. In most cases, the null model was the best-fitting model, or the model included factors that showed only anecdotal evidence in the posterior summaries. The only exception was the model including both a categorical and continuous predictor for addition in younger adults (*P(M)* = 0.33, *P(M|*data*)* = 0.59, *BF*_*M*_ = 2.85, *BF*_*10*_ = 3.25), which showed moderate evidence for a categorical carry effect (*BF*_*10*_ = 3.56) and anecdotal evidence for the unit sum (*BF*_*10*_ = 1.67) in the left MFG (channel 4). Given this weak pattern of results, we refrain from interpreting neural processing characteristics related to carry and borrow effects.

### Brain-behavior correlations

In an exploratory analysis, brain-behavior correlations were calculated between cognitive measures (processing speed, working memory, verbal/non-verbal intelligence), behavioral carry/borrow effects (ACC, RT), and neural carry/borrow effects for each age group (see Tables G-L in S1 File). Concerning behavioral effects, higher verbal intelligence was associated with larger borrow effects (ACC) in children and younger adults, higher verbal intelligence and working memory (ACC) with smaller carry or borrow effects (RT) in younger adults, and higher non-verbal intelligence and processing speed with larger carry or borrow effects (ACC) in older adults. Concerning neural effects, higher verbal intelligence and working memory (ACC) were associated with smaller borrow effects in the right IFG in children, higher working memory (ACC) with smaller carry effects in the left SMG and with smaller borrow effects in the left IFG in younger adults, higher non-verbal intelligence with larger borrow effects in the right IPS in older adults, and higher processing speed with larger borrow effects in the left MFG and right AG in older adults. Concerning the relation between behavioral (RT) and neural effects, carry effects were positively associated in the right MFG and left IPS in younger adults, borrow effects in the left IFG and MFG in younger adults, and carry effects in the bilateral MFG in older adults.

## Discussion

This study aimed to evaluate the gradual fronto-parietal shift for lifespan development of arithmetic. Arithmetic was found to be processed in the fronto-parietal network, and activation within this network increased with task difficulty, as indicated by arithmetic complexity and operation, but did not substantially vary with age. This suggests that solving complex two-digit arithmetic requires procedural calculation steps and place-value computation, being subserved by the fronto-parietal network independent of age. Due to the high task demands, age-related changes in brain activation – such as the gradual developmental shift from frontal to parietal activation or general developmental changes – were not observed, that might be limited to single-digit arithmetic. Nevertheless, place-value computation, as indicated by the carry and borrow effects, became more efficient across the whole lifespan.

Arithmetic processing relied on the fronto-parietal network in children as well as in younger and older adults [73–75], confirming the involvement of the magnitude network related to procedural strategies in arithmetic processing [76,77], regardless of age. Unexpectedly, no evidence was found for a developmental fronto-parietal shift across the lifespan [78,79], as bilateral frontal activation in the IFG and MFG, as well as bilateral parietal activation in the IPS and SMG, was similar for all age groups. Although children required more time to solve arithmetic problems than adults, they did not rely more on frontal brain resources [80]. Nevertheless, exploratory results indicated that frontal activation was indeed higher and more prolonged in children than in adults, corroborating behavioral performance and partially supporting the developmental fronto-parietal shift in terms of a decrease in frontal activation during development [78,81]. No strong evidence was found for differences in accuracy, suggesting that children might be able to solve two-digit arithmetic problems but need more time to do so. Accordingly, no qualitative differences in brain activation patterns were observed, and children relied on the same frontal brain resources as adults, reflecting the high task demands of two-digit arithmetic that might have masked age-related differences.

Complementary, the fronto-parietal shift was not observed in terms of an increase in parietal activation during development [78,80,82–85]. Instead, the IPS was similarly – or, according to the exploratory results, even more – active during arithmetic processing in children compared to adults. This might point to a more intensive recruitment of neural resources in children when solving arithmetic problems that are not yet automatized at this age and require stepwise manipulations of numbers within the place-value system [86], potentially still partially involving counting strategies [87]. Hence the functional specialization of the IPS during development does not generalize from single-digit basic number magnitude processing [80,83–85] to more complex arithmetic involving multi-digit numbers. Thus, the role of the IPS in arithmetic might depend more on the operations and complexity involved than on age [87,88], or it may emerge earlier in development, prior to grade 3, rather than in parallel with the decrease in frontal activation [82]. Taken together, evidence for a developmental fronto-parietal shift in brain activation during two-digit arithmetic is limited and, if present at all, only partially supported for frontal activation decrease when comparing children and adults. Consequently, the developmental fronto-parietal shift might not reflect a shift toward functional specialization in the IPS, but rather increasing automatization in the AG and SMG associated with changes in strategy use from procedural calculation to fact retrieval [89,90]. This might explain why the developmental fronto-parietal shift only applies to single-digit arithmetic, which increasingly relies on fact retrieval across the lifespan, but not to two-digit arithmetic, which mostly relies on procedural strategies [91,92].

Regarding aging, older adults did not show deficits in arithmetic performance compared to younger adults and also exhibited similar activation within the fronto-parietal network. This finding suggests stability of arithmetic processing in the adult brain rather than age-related changes during adulthood. Nevertheless, this seemingly uniform picture may obscure underlying age-related changes occurring in opposite directions: Within the magnitude network supporting arithmetic processing and procedural strategies [76,77,93], older adults may be more proficient in domain-specific processes [94,95], leading to higher functional specialization and reduced frontal activation. At the same time, they may exhibit deficits in domain-general processes [96], necessitating compensatory mechanisms and increased frontal activation. Given the limited spatial resolution of fNIRS, such differential activation within distinct subregions of the IFG and MFG cannot be resolved; this limitation might be addressed in future studies using fMRI. By contrast, the stability of IPS activation during aging points to a functional specialization for number processing and procedural calculation strategies [74,89,93] that might be established after formal education and maintained throughout adulthood without further refinement or age-related deterioration. Given this evidence, we conclude that the gradual developmental fronto-parietal shift does not continue during adulthood for two-digit arithmetic.

Activation within the fronto-parietal network increased with arithmetic complexity in a similar manner across all age groups. Solving addition problems with carrying and subtraction problems with borrowing took longer and was more error-prone than solving problems without carrying or borrowing, reflecting increased task difficulty and replicating previous findings [for a review see 97]. The carry and borrow effects were associated with increased frontal activation in the bilateral IFG and MFG, as expected, reflecting higher domain-general demands such as working memory [63,98]. Moreover, the effects were also associated with increased parietal activation in the bilateral IPS, which is surprising given that problem size was not confounded in the current study [as in 99, 100]. This parietal activation might reflect increased domain-specific demands such as place-value processing [101], as carry and borrow operations require computations across place values [97]. Previously, IPS activation has mainly been reported for continuous rather than categorical processing characteristics of the carry effect [102]. Unfortunately, we were not able to differentiate between these processing characteristics in the current study; thus, the underlying processes of the carry and borrow effects require further investigation.

The carry and borrow effects were larger in children than in adults, and larger in younger than in older adults. This decrease in carry and borrow effects across the lifespan replicates previous behavioral research [94,103,104] and suggests that carry and borrow operations are particularly challenging during early stages of math education, whereas increasing lifetime experience may lead to higher proficiency in the arithmetic procedures required for place-value computation. Interestingly, this developmental trajectory of the effects was not consistently accompanied by changes in the underlying processing characteristics or brain activation. This might be explained by the increased frontal control demands associated with procedural calculation strategies across all age groups [93], which might obscure smaller age-related differences. As an exception, frontal activation in posterior parts of the right MFG was associated with carry and borrow effects only in adults but not in children. This result was confirmed by exploratory analyses and further extended to the right IPS and SMG. This pattern points towards a differential involvement of these regions in dealing with task complexity in more proficient adults compared to less proficient children, corroborating previous findings in younger children [82] as well as in high- and low-performing individuals [63]. These findings suggest that the developmental fronto-parietal shift does not apply to arithmetic complexity effects.

Differences between arithmetic operations were observed at both the behavioral and neural levels as well. Subtraction was found to be more difficult than addition, as indicated by longer response times and delayed, increased activation in the bilateral IFG, MFG, and IPS. The behavioral operation effect was larger in children than in adults, echoing that inverse arithmetic operations are more difficult to acquire and are learned later [97]. Nevertheless, neural activation associated with the operation effect in the bilateral IFG, MFG and IPS did not vary between age groups. Thus, neural activation differences between addition and subtraction may be more likely to occur in subcortical rather than cortical brain regions [99], which lie outside the scope of fNIRS. Furthermore, the carry effect was found to consist of both categorical and continuous processing characteristics, whereas the borrow effect was found to be primarily categorical – independent of age. This is mostly in line with previous research [103,105,106], with the exception that children in this age range have previously been shown to exhibit categorical processing only for the carry effect [103,106], which may be attributable to the use of a production paradigm in the current study instead of a forced-choice paradigm. Interestingly, accuracy data revealed a pattern opposite to that observed for response times: categorical processing for the carry effect and continuous processing for the borrow effect – again independent of age. These results indicate that differences in processing characteristics between carry and borrow effects are driven by the arithmetic operation and outcome measure rather than by development. Taken together, differences in performance between addition and subtraction, along with differences in processing characteristics between carry and borrow effects, highlight that arithmetic processing depends on the specific arithmetic operation involved, such that subtraction differs from addition despite their mathematical similarity across all age groups [97].

As the first neuroimaging study to investigate the lifespan development of arithmetic, this study has two main limitations. First, comparisons between age groups may not be specific to arithmetic processing. In line with previous findings, processing speed and working memory also undergo developmental changes across the lifespan [103,107,108]. Therefore, differences in response times in the arithmetic task could reflect general age-related changes in processing speed; however, these changes are unlikely to explain differences in the arithmetic effects. Furthermore, because recruitment strategies differ between age groups, sampling biases cannot be entirely ruled out. Nevertheless, the observed differences in intelligence ran counter to our expectations (given that younger adults are typically university students with higher levels of intelligence). Consequently, intelligence was unlikely to be confounded with development but may instead have led to an underestimation of age-related effects. Second, the methodological choice of fNIRS was based on its suitability for use across different age groups [109] and its feasibility for an arithmetic production paradigm [110]. The disadvantages of fNIRS, however, may have contributed to the null results regarding age-related differences. In particular, it remains unclear to what extent brain activation measured with fNIRS can be reliably compared across age groups, given that the signal may be influenced by age‐related differences in head size, skull properties, and cerebral as well as skin blood flow, and that fNIRS measures functional but not anatomical data. Future studies combining fNIRS with short-channel measurements or fMRI could help improve comparability. Furthermore, due to its limited spatial and depth resolution, fNIRS is restricted to cortical regions and cannot capture activation in subcortical regions or fine-grained local differences within cortical regions. In addition, arithmetic processing relies on a distributed network that includes regions such as the dorsomedial prefrontal cortex, the cingulate gyrus, and the insula [74,89,93]. Since our fNIRS probeset only covered lateral frontal and parietal areas, age-related differences might still occur in other brain regions.

## Conclusions

Based on this first neuroimaging study investigating arithmetic development across the lifespan, we conclude that processing two-digit arithmetic is remarkably stable within the frontal and parietal cortex. The fronto-parietal network supporting arithmetic processing is more engaged as task demands increase due to arithmetic complexity and operation. Although these complexity and operation effects decrease over development, their associated patterns of brain activation remain relatively unchanged. Therefore, the present findings suggest that the developmental fronto-parietal shift may not generalize from single-digit to two-digit arithmetic and arithmetic effects. While there is some evidence for a decrease in frontal activation from childhood to adulthood, no corresponding increase in parietal activation was observed, and no comparable age-related changes were evident in adulthood. During aging, arithmetic performance showed no evidence of decline, and place-value processing may even be enhanced through life-long experience. Hence, arithmetic skills acquired in school are maintained and used throughout life, distinguishing arithmetic from many other cognitive domains that typically exhibit age-related declines [103].

## Supporting information

S1 File Additional tables and figures. The Supplementary Material includes information on the exclusion of participants and trials, the behavioral results for accuracy, time series graphs for fNIRS data, results tables for confirmatory and exploratory fNIRS analyses, and brain-behavior correlations.
(PDF)
