# Supplementary material for: No developmental fronto-parietal shift in brain activation during mental arithmetic across the lifespan: A registered report
Source: PLoS One. 2026 Aug 5;21(8):e0353139. doi: 10.1371/journal.pone.0353139 (PMC13440826; doi:10.1371/journal.pone.0353139)
Supplement: Supplementary file 1 [file pone.0353139.s001.pdf]

SUPPLEMENTARY MATERIAL S1

***Paper*:** **No developmental fronto-parietal shift of brain activation during mental arithmetic across the lifespan: A Registered Report**

***Authors*:** **Christina Artemenko & Mine Avcil**

Table A. *Exclusion of participants and trials.*

Tasks	children	younger adults	older adults
***N* sample size**	60	62	61
**exclusion from all analysis**			
demographics (age, handedness, language, health)	0	0	0
cognition (MoCA score < 26)	-	-	5
missing data (missing in arithmetic > 50%)	4	2	0
accuracy (ACC in arithmetic < 50%)	1	0	1
group outlier (RT deviating from *Median* ± 3 *MAD*)	0	1	3
**exclusion from fNIRS**			
missing data (missing in fNIRS > 50%)	0	0	1
noisy channels (fNIRS > 5) *	6	1	0
**exclusion from processing speed**			
missing data in processing speed	1	23	4
**exclusion from working memory**			
missing data in working memory	11	0	5
accuracy (ACC in working memory < 33%)	5	3	1
**exclusion from intelligence**			
missing data in intelligence	0	0	1
**exclusion from math anxiety**			
missing data in math anxiety	2	0	1
**trial exclusion from arithmetic and fNIRS**			
incorrectly solved	14.50%	9.84%	8.98%
RT < 200 ms	0.00%	0.00%	0.00%
RT outlier	3.37%	3.10%	4.27%
RT duration outlier	3.91%	2.46%	3.85%
**trial exclusion from working memory**			
incorrectly solved	28.66%	9.23%	17.97%
RT < 200 ms	0.03%	0.02%	0.02%
RT outlier	0.57%	4.25%	3.29%

Note. The assessment of working memory by the n-back task turned out to be inappropriate for children in this age (high exclusion rate due to drop out and low accuracy) and partly also for older adults (unfamiliar with computerized tasks). *The criterion for noisy channels was increased from 3 to 5, as noisy channels were not interpolated but excluded (deviating from the registered report protocol).

Behavioral results for accuracy

For the arithmetic task, the best model of ACC included the main effects of operation, complexity and age group, and the two-way interaction of age group with complexity (P(M) = 0.053, P(M|data) = 0.342, BF_M = 9.36, BF_{10} > 100, error% = 10.289). Extreme evidence was found for the main effects of operation (BF_{incl} > 100) and complexity (BF_{incl} > 100) indicating that solving subtraction is more prone to errors than solving addition and that problems with the carry or borrow operation are more prone to errors than problems without the carry or borrow operation. Evidence for the main effect of age group was only moderate (BF_{incl} = 4.69) and for the interaction between age group and complexity anecdotal (BF_{incl} = 1.62).

For addition, the regressions on categorical (carry) and continuous predictors (unit sum) revealed the following results concerning ACC (see Table B): For addition, the best models included only carry in children, younger and older adults, however, the carry effect was inconclusive in younger adults (BF_{incl} = 2.34). For subtraction, the best models included only unit difference in children, younger and older adults, however, the effect of unit difference was inconclusive older adults (BF_{incl} = 1.60).

Table B. *Behavioral processes underlying carry and borrow effects (Bayesian Regressions).*

Table B1. *Model Comparison (Bayesian Regressions).*

	addition					subtraction				
	**model**	***P(M)***	***P(M|data)***	***BF*** BF_M	BF_{10}	**model**	***P(M)***	***P(M|data)***	BF_M	BF_{10}
					**children**					
**RT**	carry + unit sum	0.333	0.993	> 100	> 100	borrow + unit difference	0.333	0.581	2.77	> 100
**ACC**	carry	0.167	0.417	3.58	> 100	unit difference	0.167	0.780	17.77	> 100
					**younger adults**					
**RT**	carry + unit sum	0.333	0.999	> 100	> 100	borrow	0.167	0.845	27.17	> 100
**ACC**	carry	0.167	0.357	2.14	> 100	unit difference	0.167	0.671	10.19	> 100
					**older adults**					
**RT**	carry + unit sum	0.333	0.780	7.10	> 100	borrow	0.167	0.769	16.66	> 100
**ACC**	carry	0.167	0.591	7.22	> 100	unit difference	0.167	0.362	2.84	10.97

Table B2. *Posterior summaries of coefficients (Bayesian Regressions).*

			**addition**				
			**children**				
**Measure**	**Coefficient**	***P*(incl)**	***P*(incl|data)**	***BF***	***M***	***SD***	**95% Credible Interval**
RT	*intercept*	1.000	1.000	1.00	8020	91	[7841, 8191]
	carry	0.500	0.998	> 100	1683	414	[867, 2420]
	unit sum	0.500	0.995	> 100	180	47	[89, 264]
ACC	*intercept*	1.000	1.000	1.00	0.90	0.01	[0.89, 0.91]
	carry	0.500	0.751	3.01	-0.04	0.03	[-0.09, 0.00]
	unit sum	0.500	0.583	1.40	0.00	0.00	[-0.01, 0.00]
			**younger adults**				
**Measure**	**Coefficient**	***P*(incl)**	***P*(incl|data)**	**BF_{incl}**	***M***	***SD***	**95% Credible Interval**
RT	*intercept*	1.000	1.000	1.00	3216	33	[3154, 3284]
	carry	0.500	1.000	> 100	701	145	[432, 996]
	unit sum	0.500	1.000	> 100	81	16	[51, 115]
ACC	*intercept*	1.000	1.000	1.00	0.93	0.00	[0.92, 0.93]
	carry	0.500	0.701	2.34	-0.03	0.02	[-0.06, 0.00]
	unit sum	0.500	0.643	1.80	0.00	0.00	[-0.01, 0.00]
			**older adults**				
**Measure**	**Coefficient**	***P*(incl)**	***P*(incl|data)**	**BF_{incl}**	***M***	***SD***	**95% Credible Interval**
RT	*intercept*	1.000	1.000	1.00	2999	35	[2931, 3067]
	carry	0.500	0.781	3.57	296	206	[0, 602]
	unit sum	0.500	0.999	> 100	-1	8	[-27, 16]
ACC	*intercept*	1.000	1.000	1.00	0.95	0.01	[0.94, 0.96]
	carry	0.500	0.922	11.79	-0.03	0.02	[-0.06, 0.00]
	unit sum	0.500	0.402	0.67	0.00	0.00	[0.00, 0.00]
			**subtraction**				
			**children**				
**Measure**	**Coefficient**	***P*(incl)**	***P*(incl|data)**	**BF_{incl}**	***M***	***SD***	**95% Credible Interval**
RT	*intercept*	1.000	1.000	1.00	9761	126	[9486, 9979]
	borrow	0.500	0.990	94.95	2277	694	[876, 3335]
	unit difference	0.500	0.591	1.45	-65	70	[-208, 0]
ACC	*intercept*	1.000	1.000	1.00	0.86	0.01	[0.85, 0.88]
	borrow	0.500	0.220	0.28	0.00	0.01	[-0.03, 0.04]
	unit difference	0.500	0.999	> 100	0.01	0.00	[0.01, 0.01]
			**younger adults**				
**Measure**	**Coefficient**	***P*(incl)**	***P*(incl|data)**	**BF_{incl}**	***M***	***SD***	**95% Credible Interval**
RT	*intercept*	1.000	1.000	1.00	4064	47	[3969, 4155]
	borrow	0.500	1.000	> 100	1319	121	[1079, 1578]
	unit difference	0.500	1.000	0.18	-1	8	[-27, 16]
ACC	*intercept*	1.000	1.000	1.00	0.89	0.01	[0.88, 0.91]
	borrow	0.500	0.329	0.49	0.00	0.02	[-0.07, 0.03]
	unit difference	0.500	0.952	20.03	0.01	0.00	[0.00, 0.01]
			**older adults**				
**Measure**	**Coefficient**	***P*(incl)**	***P*(incl|data)**	**BF_{incl}**	***M***	***SD***	**95% Credible Interval**
RT	*intercept*	1.000	1.000	1.00	3773	50	[3670, 3872]
	borrow	0.500	1.000	> 100	950	142	[663, 1258]
	unit difference	0.500	0.231	0.30	2.64	11.47	[-13, 40]
ACC	*intercept*	1.000	1.000	1.00	0.89	0.01	[0.88, 0.91]
	borrow	0.500	0.572	1.33	-0.02	0.02	[-0.06, 0.01]
	unit difference	0.500	0.615	1.60	0.00	0.00	[0.00, 0.01]

Figure A. *Brain activation of children during arithmetic dependent on operation and complexity (time series graph).*

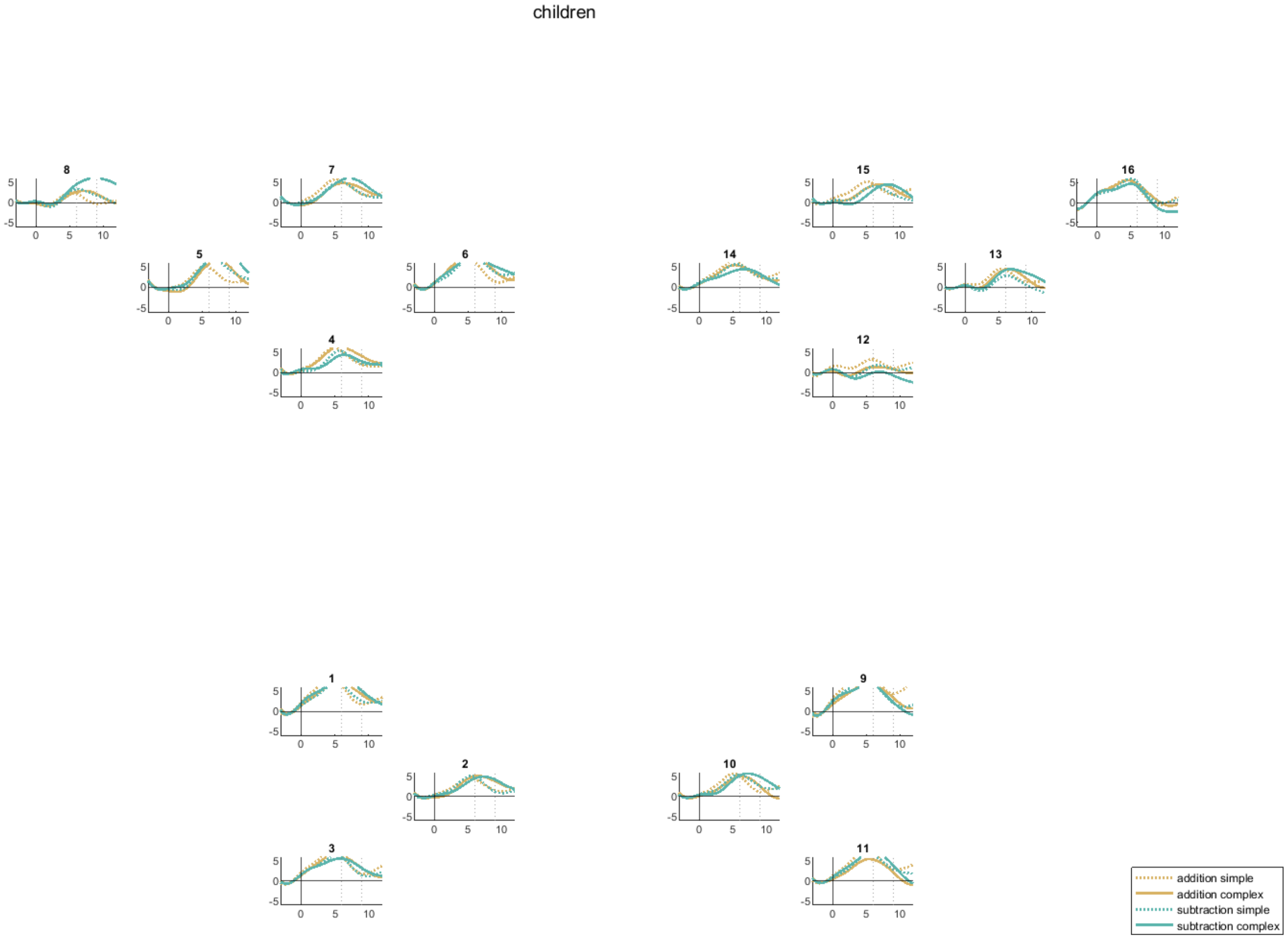

Notes. The time-series graphs per channel include all conditions (addition/subtraction simple/complex) and additionally display latencies of 6 and 9 s (by dotted grey bars).

Figure B. *Brain activation of younger adults during arithmetic dependent on operation and complexity (time series graph).*

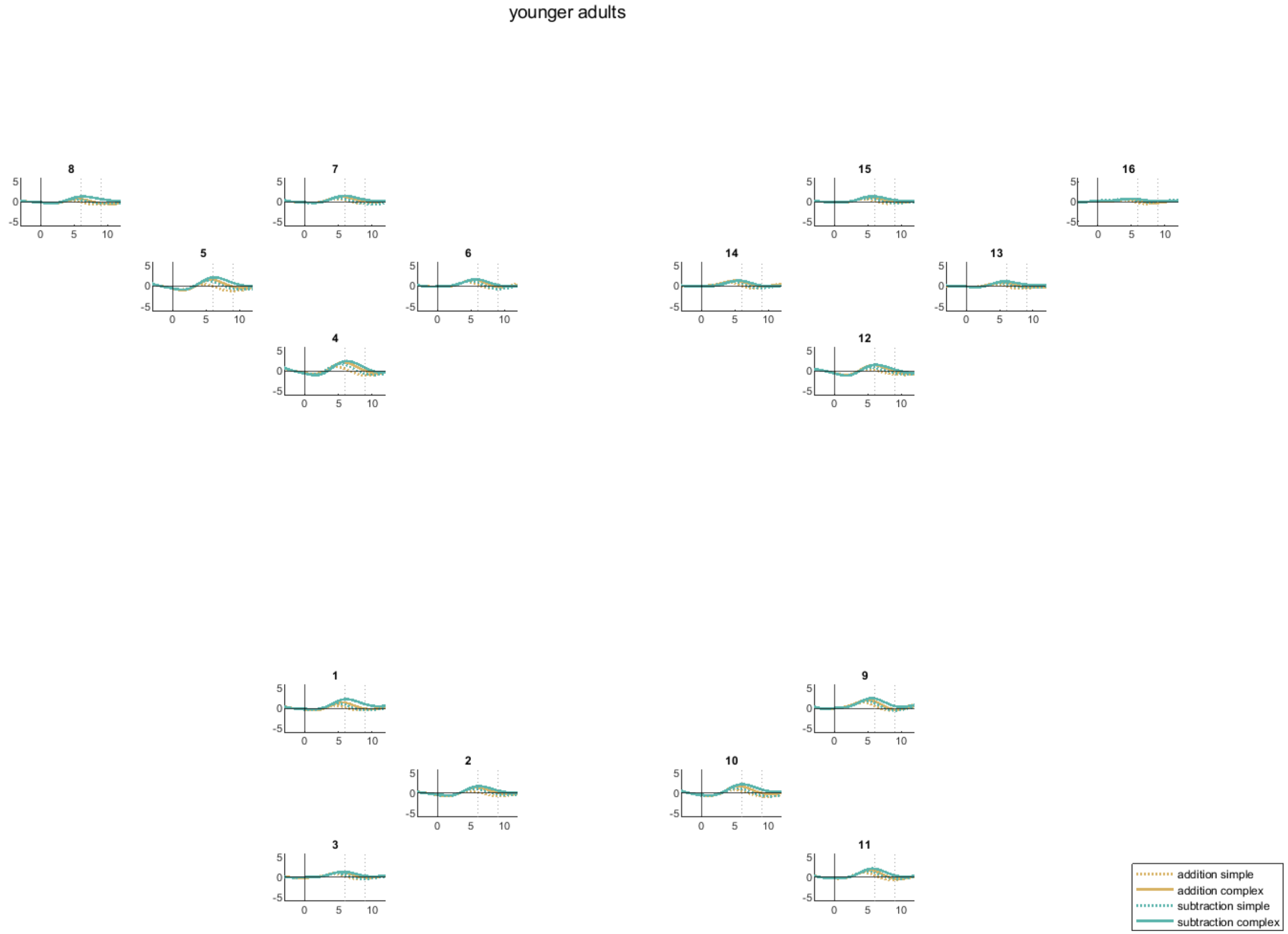

Notes. The time-series graphs per channel include all conditions (addition/subtraction simple/complex) and additionally display latencies of 6 and 9 s (by dotted grey bars).

Figure C. *Brain activation of older adults during arithmetic dependent on operation and complexity (time series graph).*

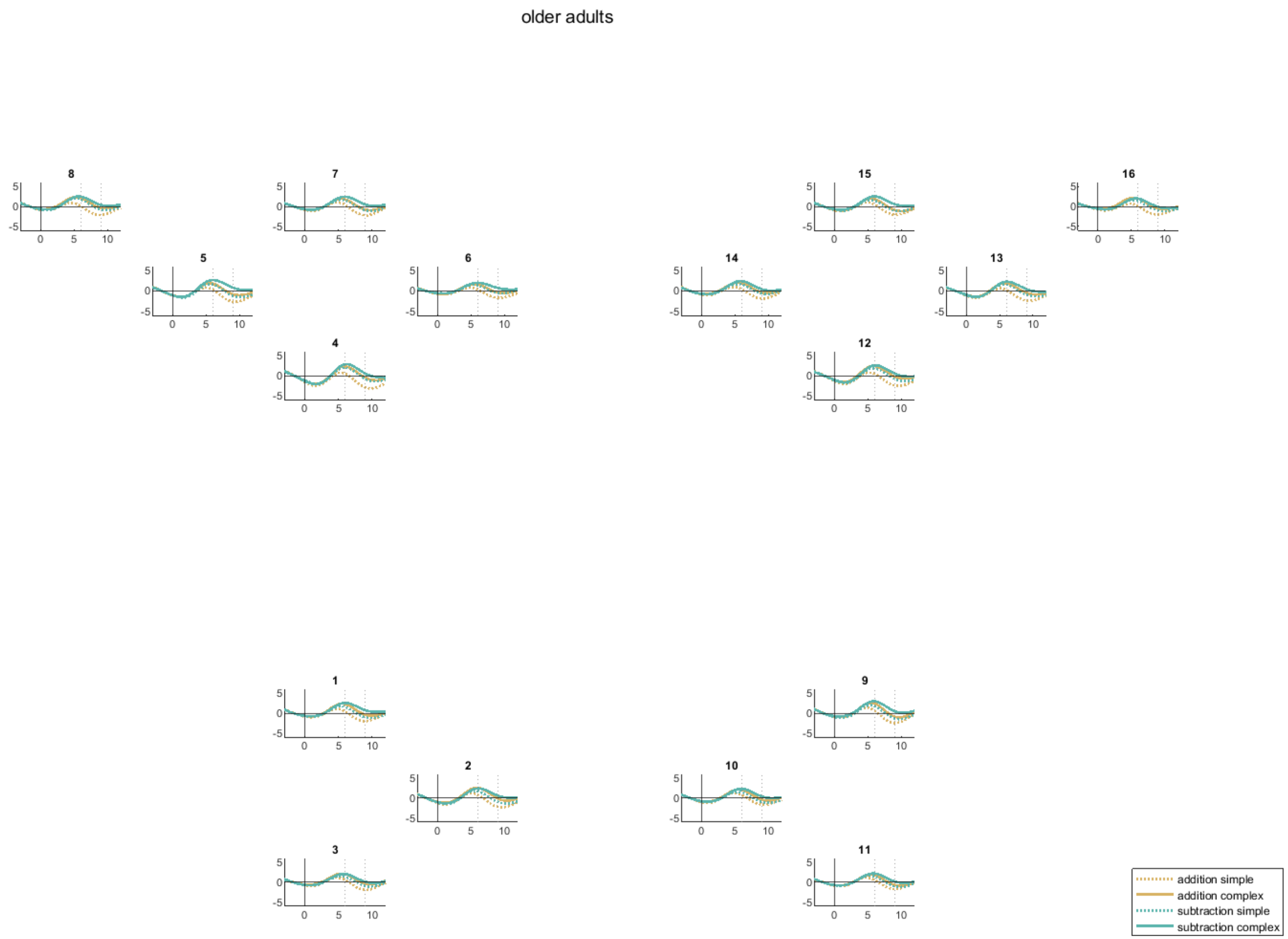

Notes. The time-series graphs per channel include all conditions (addition/subtraction simple/complex) and additionally display latencies of 6 and 9 s (by dotted grey bars).

Table C. *Brain activation during arithmetic dependent on age, operation, and complexity (based on preregistered GLM with HRF peak at 6 s).*

brain area	hemis-phere	ch	MNI coordinates			age		operation		complexity		age × operation		age × complexity		operation × complexity		age × operation × complexity	
			x	y	z	BF_{incl}	BF_{excl}	BF_{incl}	BF_{excl}	BF_{incl}	BF_{excl}	BF_{incl}	BF_{excl}	BF_{incl}	BF_{excl}	BF_{incl}	BF_{excl}	BF_{incl}	BF_{excl}
IFG	left	5	-54.989	21.577	28.224	0.51	1.96	0.29	3.50	0.13	8.00	0.14	7.09	0.05	18.52	0.15	6.62	0.15	6.76
		8	-54.192	35.192	10.404	0.73	1.37	0.10	9.62	0.11	8.85	0.09	11.11	0.09	11.36	0.57	1.75	0.10	9.90
	right	13	56.431	21.530	28.382	0.05	20.41	0.34	2.94	0.12	8.13	**5.77**		0.16	6.17	1.59		0.00	> 100
		16	56.186	34.089	11.569	0.50	2.01	0.12	8.62	0.16	6.17	0.06	16.67	0.07	14.29	0.23	4.39	0.09	11.76
MFG	left	4	-52.981	9.441	43.288	0.36	2.82	0.22	4.52	0.21	4.67	1.33		0.77	1.30	0.15	6.54	1.79	
		6	-40.683	22.891	50.628	0.89	1.13	0.07	14.93	0.09	11.36	0.22	4.59	0.38	2.67	0.27	3.68	0.72	1.39
		7	-43.559	36.844	35.329	0.50	2.00	0.11	9.01	0.13	8.00	0.07	14.29	0.08	12.82	0.20	5.08	0.20	4.95
	right	12	54.448	8.156	43.809	**5.07**		0.15	6.49	0.14	7.35	0.16	6.33	0.07	14.49	0.70	1.43	0.11	9.09
		14	41.653	22.829	50.474	0.37	2.70	0.10	9.80	0.09	10.64	0.10	9.90	0.31	3.22	0.21	4.78	0.39	2.58
		15	45.204	35.666	36.000	0.50	2.00	0.16	6.17	0.36	2.79	0.48	2.10	0.16	6.17	0.79	1.27	0.01	> 100
IPS	left	2	-42.484	-56.425	59.293	0.29	3.42	0.16	6.33	0.44	2.28	0.35	2.89	0.09	11.63	0.77	1.31	0.52	1.93
	right	10	41.785	-56.609	59.831	0.58	1.71	0.38	2.65	0.19	5.32	0.17	5.95	0.06	17.54	0.64	1.56	0.16	6.41
AG	left	3	-45.161	-69.940	48.359	0.39	2.56	0.25	4.02	0.13	8.00	0.08	12.35	0.26	3.92	0.11	8.77	0.38	2.66
	right	11	43.703	-69.098	48.517	1.97		0.16	6.29	0.11	9.35	0.11	8.93	0.16	6.33	0.17	5.88	0.07	14.08
SMG	left	1	-56.851	-46.507	50.825	0.33	3.07	0.10	9.71	0.11	9.35	0.10	10.53	0.03	40.00	0.27	3.75	0.41	2.47
	right	9	56.900	-46.866	51.037	0.22	4.65	0.15	6.85	0.18	5.43	0.01	76.92	0.06	16.95	0.12	8.20	0.28	3.53

Notes. BF_{incl} and BF_{excl} (if BF_{incl} < 1) are given for the analysis of effects in Bayesian ANOVAs. BF_{incl} > 3 indicate evidence for an effect (marked in **bold**) and BF_{excl} > 3 indicate evidence against an effect (marked in red). ch – channel, GLM – general linear model, HRF – hemodynamic response function.

According to the registered report, the GLM should have been based on a hemodynamic response function (HRF) with a peak at 6 s. This peak latency was calculated by detecting the peak within a time window of [4 10] s for each channel, subject, and condition, and by calculating the weighted mean across all channels, subjects per group and conditions (see Figures S1, S2, S3). However, the results of the GLM showed – with two exceptions – mostly null effects (for operation, complexity, and interactions) or inconclusive effects (for age; see Table C). This indicates that the GLM did not capture the data well but fails to reflect the structure of the data. This suspicion was confirmed by exploratory analyses on mean amplitudes (see Table E) and peak latencies (see Table F). The analysis of mean amplitudes indeed reveals that more difficult arithmetic problems are associated with higher brain activation in the fronto-parietal network than simple problems (operation and complexity effects). Moreover, the analysis of peak latencies reveals that more difficult arithmetic problems are further associated with later peak latencies than simple problems in the fronto-parietal network (operation and complexity effects). This means that if the peak of the HRF is set as early as 6 s, particularly more difficult problems were not appropriately modelled in the GLM, which might be a reason for the lack of effects in this analysis. As a consequence, we chose a later peak at 9 s for the GLM which was usually used in fNIRS studies on two-digit arithmetic using a similar paradigm (Artemenko et al., 2018). The results of this GLM (see Table D) reveal operation and complexity effects similar to the analysis on amplitudes and thus seem to more appropriately capture differences between conditions in the data.

Table D. *Brain activation during arithmetic dependent on age, operation, and complexity (based on final GLM with HRF peak at 9 s).*

brain area	hemis-phere	ch	MNI coordinates			age		operation		complexity		age × operation		age × complexity		operation × complexity		age × operation × complexity	
			x	y	z	BF_{incl}	BF_{excl}	BF_{incl}	BF_{excl}	BF_{incl}	BF_{excl}	BF_{incl}	BF_{excl}	BF_{incl}	BF_{excl}	BF_{incl}	BF_{excl}	BF_{incl}	BF_{excl}
IFG	left	5	-54.989	21.577	28.224	1.92		**> 100**		**> 100**		0.09	11.36	0.24	4.12	1.77		0.12	8.13
		8	-54.192	35.192	10.404	2.26		**> 100**		**> 100**		0.18	5.68	0.17	5.88	0.53	1.87	0.10	10.10
	right	13	56.431	21.530	28.382	2.07		**> 100**		**> 100**		0.15	6.67	0.15	6.90	1.12		0.10	9.90
		16	56.186	34.089	11.569	0.25	4.00	**> 100**		**> 100**		0.41	2.46	0.40	2.51	0.36	2.79	0.10	9.71
MFG	left	4	-52.981	9.441	43.288	0.46	2.20	**> 100**		**> 100**		0.06	17.54	0.43	2.34	1.25		0.12	8.33
		6	-40.683	22.891	50.628	0.41	2.43	**> 100**		**> 100**		0.18	5.49	0.27	3.76	0.36	2.82	0.07	14.93
		7	-43.559	36.844	35.329	0.66	1.52	**> 100**		**> 100**		0.68	1.47	0.11	9.01	0.15	6.90	0.19	5.41
	right	12	54.448	8.156	43.809	0.19	5.29	**> 100**		**> 100**		0.07	13.70	**5.72**		0.15	6.49	0.47	2.13
		14	41.653	22.829	50.474	0.57	1.77	**> 100**		**> 100**		0.33	3.04	0.10	9.90	0.12	8.06	0.39	2.57
		15	45.204	35.666	36.000	0.76	1.32	**> 100**		**> 100**		0.89	1.12	0.16	6.33	0.15	6.67	0.09	11.36
IPS	left	2	-42.484	-56.425	59.293	0.19	5.24	**> 100**		**> 100**		0.16	6.33	0.12	8.70	0.33	3.06	0.22	4.65
	right	10	41.785	-56.609	59.831	0.68	1.47	**> 100**		**> 100**		0.10	9.90	0.03	32.26	0.14	7.35	0.03	34.48
AG	left	3	-45.161	-69.940	48.359	0.39	2.58	**> 100**		**> 100**		0.09	11.63	0.05	21.74	0.14	6.99	0.08	12.35
	right	11	43.703	-69.098	48.517	0.61	1.64	**> 100**		**> 100**		0.08	12.82	0.11	9.26	0.13	7.52	0.19	5.32
SMG	left	1	-56.851	-46.507	50.825	0.38	2.67	**> 100**		**> 100**		0.03	33.33	0.23	4.39	0.18	5.62	0.10	10.00
	right	9	56.900	-46.866	51.037	0.53	1.90	**> 100**		**> 100**		0.79	1.26	0.11	9.09	0.15	6.71	0.13	7.63

Notes. BF_{incl} and BF_{excl} (if $BF_{incl} < 1$) are given for the analysis of effects in Bayesian ANOVAs. $BF_{incl} > 3$ indicate evidence for an effect (marked in **bold**) and $BF_{excl} > 3$ indicate evidence against an effect (marked in red). ch – channel, GLM – general linear model, HRF – hemodynamic response function.

These are the main results presented in the paper (see Table 2 in the manuscript, for more details see Table D in the Supplementary Material).

Table E. *Brain activation during arithmetic dependent on age, operation, and complexity (based on mean amplitudes).*

brain area	hemis-phere	ch	MNI coordinates			age		operation		complexity		age × operation		age × complexity		operation × complexity		age × operation × complexity	
			x	y	z	BF_{incl}	BF_{excl}	BF_{incl}	BF_{excl}	BF_{incl}	BF_{excl}	BF_{incl}	BF_{excl}	BF_{incl}	BF_{excl}	BF_{incl}	BF_{excl}	BF_{incl}	BF_{excl}
IFG	left	5	-54.989	21.577	28.224	**65.11**		**> 100**		**> 100**		0.06	16.39	0.20	5.03	0.15	6.90	0.07	14.49
		8	-54.192	35.192	10.404	0.48	2.10	**> 100**		**> 100**		0.04	22.73	0.08	12.99	0.13	7.63	0.46	2.16
	right	13	56.431	21.530	28.382	1.32		0.50	2.02	**> 100**		1.05		0.11	9.35	0.13	7.63	0.26	3.83
		16	56.186	34.089	11.569	**11.39**		0.42	2.37	1.35		0.09	10.64	0.42	2.38	0.51	1.97	0.14	6.99
MFG	left	4	-52.981	9.441	43.288	**3.51**		0.49	2.05	**> 100**		2.05		0.55	1.82	1.29		0.08	12.35
		6	-40.683	22.891	50.628	**> 100**		**3.42**		**> 100**		0.09	11.11	0.08	12.35	0.23	4.33	0.09	11.24
		7	-43.559	36.844	35.329	**> 100**		**3.25**		**> 100**		0.29	3.42	0.37	2.70	0.15	6.90	0.16	6.25
	right	12	54.448	8.156	43.809	0.14	6.99	0.20	4.93	**6.24**		1.56		**27.62**		0.12	8.20	0.17	6.02
		14	41.653	22.829	50.474	**> 100**		0.52	1.92	**> 100**		0.63	1.59	**9.15**		0.32	3.15	0.08	12.20
		15	45.204	35.666	36.000	2.24		0.41	2.42	**34.52**		2.30		2.09		0.15	6.80	0.06	16.67
IPS	left	2	-42.484	-56.425	59.293	**5.99**		1.15		**> 100**		0.12	8.20	0.26	3.79	0.43	2.34	0.21	4.83
	right	10	41.785	-56.609	59.831	**4.17**		**3.19**		**> 100**		0.09	10.87	**5.34**		0.12	8.20	0.05	21.28
AG	left	3	-45.161	-69.940	48.359	**> 100**		0.16	6.37	**> 100**		0.18	5.49	0.21	4.81	0.15	6.58	0.11	8.93
	right	11	43.703	-69.098	48.517	**> 100**		**9.74**		**33.91**		0.07	13.51	1.53		0.19	5.29	0.31	3.19
SMG	left	1	-56.851	-46.507	50.825	**> 100**		**3.42**		**> 100**		0.10	10.00	0.11	9.26	0.13	7.87	0.14	7.19
	right	9	56.900	-46.866	51.037	**> 100**		0.26	3.85	**8.76**		1.06		**21.91**		0.14	7.04	0.11	9.17

Notes. BF_{incl} and BF_{excl} (if $BF_{incl} < 1$) are given for the analysis of effects in Bayesian ANOVAs. $BF_{incl} > 3$ indicate evidence for an effect (marked in **bold**) and $BF_{excl} > 3$ indicate evidence against an effect (marked in red). ch – channel.

In an exploratory analysis, mean amplitudes were calculated in the time window [4 10] s and baseline-corrected according to 3 s before stimulus onset (see Table E). Evidence for a complexity effect indicated higher activation in bilateral frontal (IFG and MFG) and parietal (IPS, AG and SMG) brain regions for complex arithmetic with carrying/borrowing compared to simple arithmetic without carrying/borrowing. Evidence for an operation effect indicated higher activation in the left frontal (IFG, MFG), and parietal (right IPS, right AG and left SMG) brain regions for subtraction as compared to addition. Evidence for an age effect indicated higher activation in the bilateral fronto-parietal network in children compared to younger and older adults. Moreover, evidence for interactions of age with complexity was found: Younger adults showed larger complexity effects than children in the right MFG (channels 12, $BF_{10} = 8.92$; channel 14, $BF_{10} = 7.29$), right IPS (channel 10, $BF_{10} = 7.90$) and right SMG (channel 9, $BF_{10} = 19.63$). These results confirm the model-based results (see Table D).

Table F. *Brain activation during arithmetic dependent on age, operation, and complexity (based on peak latency).*

brain area	hemis-phere	ch	MNI coordinates			age		operation		complexity		age × operation		age × complexity		operation × complexity		age × operation × complexity	
			x	y	z	BF_{incl}	BF_{excl}	BF_{incl}	BF_{excl}	BF_{incl}	BF_{excl}	BF_{incl}	BF_{excl}	BF_{incl}	BF_{excl}	BF_{incl}	BF_{excl}	BF_{incl}	BF_{excl}
IFG	left	5	-54.989	21.577	28.224	**18.96**		**99.18**		**> 100**		0.21	4.81	0.08	12.66	0.15	6.58	0.15	6.85
		8	-54.192	35.192	10.404	**33.21**		**> 100**		**> 100**		0.09	10.87	0.29	3.51	0.13	7.63	1.33	
	right	13	56.431	21.530	28.382	1.78		**10.53**		**11.78**		0.10	10.10	0.14	7.25	0.13	7.87	0.17	5.75
		16	56.186	34.089	11.569	**65.50**		1.02		0.59	1.70	0.47	2.11	0.11	8.93	0.26	3.79	0.10	9.71
MFG	left	4	-52.981	9.441	43.288	0.40	2.53	**5.59**		**11.17**		0.14	6.94	0.13	7.63	0.21	4.85	0.24	4.15
		6	-40.683	22.891	50.628	0.39	2.54	**> 100**		**> 100**		0.15	6.62	0.09	10.64	0.12	8.06	0.12	8.26
		7	-43.559	36.844	35.329	**4.12**		**26.85**		**3.65**		0.11	9.35	0.13	7.94	0.19	5.32	0.33	3.02
	right	12	54.448	8.156	43.809	**30.76**		0.64	1.56	**> 100**		0.28	3.62	0.42	2.39	0.44	2.28	0.07	14.29
		14	41.653	22.829	50.474	2.52		0.54	1.85	**3.18**		0.24	4.13	0.10	10.31	0.23	4.37	0.08	12.82
		15	45.204	35.666	36.000	**12.61**		**20.36**		**13.34**		1.28		0.10	9.71	0.53	1.90	1.91	
IPS	left	2	-42.484	-56.425	59.293	1.96		**5.36**		**> 100**		0.40	2.53	0.08	12.66	0.14	7.30	0.07	13.51
	right	10	41.785	-56.609	59.831	**3.08**		**> 100**		**> 100**		0.16	6.45	0.07	14.71	0.22	4.48	0.10	10.31
AG	left	3	-45.161	-69.940	48.359	1.43		0.36	2.75	**70.65**		0.21	4.67	0.18	5.62	0.15	6.54	0.15	6.54
	right	11	43.703	-69.098	48.517	0.33	2.99	0.32	3.12	**78.64**		0.09	11.49	0.13	7.81	0.17	5.78	0.08	12.66
SMG	left	1	-56.851	-46.507	50.825	0.69	1.44	0.19	5.35	**15.93**		0.18	5.49	0.06	17.54	0.24	4.18	0.12	8.20
	right	9	56.900	-46.866	51.037	0.26	3.85	0.12	8.40	**16.29**		0.67	1.48	0.23	4.29	0.21	4.83	0.09	10.87

Notes. BF_{incl} and BF_{excl} (if BF_{incl} < 1) are given for the analysis of effects in Bayesian ANOVAs. BF_{incl} > 3 indicate evidence for an effect (marked in **bold**) and BF_{excl} > 3 indicate evidence against an effect (marked in red). ch – channel.

In an exploratory analysis, peak latency was determined for each subject, channel and condition by averaging all trials of a certain condition and detecting the maximum amplitude (peak) in the time window [4 10] s. Evidence for a complexity effect indicated a later peak of brain activation in bilateral frontal (IFG and MFG) and parietal (IPS, AG and SMG) brain regions for complex arithmetic with carrying/borrowing compared to simple arithmetic without carrying/borrowing. Evidence for an operation effect indicated a later peak of brain activation in bilateral frontal (IFG, MFG), and parietal (IPS) brain regions for subtraction as compared to addition. Evidence for an age effect indicated a later peak of brain activation in children than younger adults in bilateral IFG (channels 5, 16) and right MFG (channel 12), a later peak in older than younger adults in bilateral IFG (channels 5, 16), and an earlier peak in older than younger adults in left IFG (channel 8), bilateral MFG (channels 7, 12, 15), and right IPS (channel 10). These results for complexity and operation effects suggest the use of a later peak of the HRF in the model-based analysis to detect condition-related differences in brain activation (see Table D vs. C).

Table G. *Brain-behavior-correlations of the carry effect in children.*

variable / brain region		ch		processing speed (score)	working memory (ACC)	verbal intelligence (score)	non-verbal intelligence (score)	carry effect (ACC)	carry effect (RT)
ACC			*n*	54	39	55	55		
			r	.152	.093	.078	.076		
			BF_{10}	0.31	0.23	0.20	0.20		
RT			*n*	54	39	55	55		
			r	-.167	-.249	.041	-.032		
			BF_{10}	0.35	0.61	0.18	0.17		
IFG	left	5	*n*	34	27	34	34	34	34
			r	.183	-.107	-.116	-.145	.146	.027
			BF_{10}	0.36	0.27	0.26	0.29	0.30	0.22
		8	*n*	37	27	37	37	37	37
			r	.233	-.232	-.042	-.044	.037	.095
			BF_{10}	0.52	0.46	0.21	0.21	0.21	0.24
	right	13	*n*	39	29	39	39	39	39
			r	-.072	-.402	.077	.165	.040	.130
			BF_{10}	0.22	2.14	0.22	0.32	0.21	0.27
		16	*n*	36	27	36	36	36	36
			r	.073	-.231	-.036	.075	-.009	.236
			BF_{10}	0.23	0.45	0.21	0.23	0.21	0.52
MFG	left	4	*n*	36	27	36	36	36	36
			r	.251	-.323	.109	.028	.017	.133
			BF_{10}	0.59	0.86	0.25	0.21	0.21	0.28
		6	*n*	36	28	36	36	36	36
			r	-.062	-.311	-.227	-.135	-.150	.229
			BF_{10}	0.22	0.81	0.49	0.28	0.30	0.50
		7	*n*	35	27	35	35	35	35
			r	.111	.023	.105	-.047	-.018	.107
			BF_{10}	0.26	0.24	0.25	0.22	0.21	0.25
	right	12	*n*	36	27	36	36	36	36
			r	.093	-.225	.105	.198	.007	.191
			BF_{10}	0.24	0.44	0.25	0.40	0.21	0.38
		14	*n*	33	25	33	33	33	33
			r	.041	-.083	-.004	-.043	.138	.094
			BF_{10}	0.22	0.27	0.22	0.22	0.29	0.25
		15	*n*	39	29	39	39	39	39
			r	.017	-.113	.128	-.119	.011	.130
			BF_{10}	0.20	0.27	0.27	0.26	0.20	0.27
IPS	left	2	*n*	33	24	33	33	33	33
			r	.157	-.101	.049	.039	.064	.068
			BF_{10}	0.31	0.28	0.22	0.22	0.23	0.23
	right	10	*n*	38	28	38	38	38	38
			r	.061	.022	.244	-.202	-.048	.125
			BF_{10}	0.22	0.24	0.58	0.41	0.21	0.27
AG	left	3	*n*	35	25	35	35	35	35
			r	-.040	-.120	.182	-.178	-.157	-.063
			BF_{10}	0.22	0.29	0.36	0.35	0.31	0.22
	right	11	*n*	39	29	39	39	39	39
			r	.046	-.145	.061	-.152	.005	.274
			BF_{10}	0.21	0.30	0.21	0.30	0.20	0.79
SMG	left	1	*n*	38	29	38	38	38	38
			r	.154	-.098	.189	.082	-.117	.088
			BF_{10}	0.30	0.26	0.38	0.23	0.26	0.23
	right	9	*n*	39	29	39	39	39	39
			r	.004	-.269	.177	-.066	-.055	.299
			BF_{10}	0.20	0.60	0.35	0.22	0.21	1.04

Notes. BF_{10} is given for Bayesian correlations of carry/borrow effects in behavior (ACC – accuracy, RT – reaction time) and brain activation (per ch – channel, based on final GLM with HRF peak at 9 s), with $BF_{10} > 3$ indicating evidence for an effect (marked in **bold**).

Abbreviations of brain regions: IFG – inferior frontal gyrus, MFG – middle frontal gyrus, IPS –intraparietal sulcus, SMG – supramarginal gyrus, AG – angular gyrus.

Table H. *Brain-behavior-correlations of the borrow effect in children.*

variable / brain region		ch		processing speed (score)	working memory (ACC)	verbal intelligence (score)	non-verbal intelligence (score)	borrow effect (ACC)	borrow effect (RT)
ACC			*n*	54	39	**55**	55		
			r	.105	.335	**.333**	-.002		
			BF_{10}	0.22	1.62	**3.39**	0.17		
RT			*n*	54	39	55	55		
			r	-.228	-.323	.027	.018		
			BF_{10}	0.65	1.39	0.17	0.17		
IFG	left	5	*n*	34	27	34	34	34	34
			r	.031	-.026	.056	-.182	.011	-.091
			BF_{10}	0.22	0.24	0.22	0.35	0.21	0.24
		8	*n*	37	27	37	37	37	37
			r	.281	-.074	-.130	.035	-.072	-.204
			BF_{10}	0.80	0.26	0.27	0.21	0.22	0.41
	right	13	*n*	39	**29**	**39**	39	39	39
			r	-.004	**-.449**	**-.466**	-.060	-.254	.009
			BF_{10}	0.20	**3.94**	**14.92**	0.21	0.65	0.20
		16	*n*	36	27	36	36	36	36
			r	.184	-.354	-.236	-.101	-.120	.157
			BF_{10}	0.36	1.14	0.52	0.24	0.26	0.31
MFG	left	4	*n*	36	27	36	36	36	36
			r	.043	-.034	-.191	.052	.164	-.205
			BF_{10}	0.21	0.24	0.38	0.22	0.32	0.42
		6	*n*	36	28	36	36	36	36
			r	.197	.146	-.016	.111	.332	-.199
			BF_{10}	0.39	0.31	0.21	0.25	1.36	0.40
		7	*n*	35	27	35	35	35	35
			r	.362	.072	.054	.000	.091	-.109
			BF_{10}	1.88	0.25	0.22	0.21	0.24	0.25
	right	12	*n*	36	27	36	36	36	36
			r	.187	-.076	-.287	-.173	-.111	.020
			BF_{10}	0.37	0.26	0.83	0.34	0.25	0.21
		14	*n*	33	25	33	33	33	33
			r	-.053	-.239	-.286	-.130	-.163	.142
			BF_{10}	0.23	0.47	0.76	0.28	0.32	0.29
		15	*n*	39	29	39	39	39	39
			r	.356	.090	-.191	-.148	.037	-.344
			BF_{10}	2.18	0.26	0.38	0.30	0.20	1.84
IPS	left	2	*n*	33	24	33	33	33	33
			r	.332	.058	-.090	-.053	.085	-.396
			BF_{10}	1.19	0.26	0.24	0.23	0.24	2.61
	right	10	*n*	38	28	38	38	38	38
			r	.137	.092	.048	.027	-.124	-.007
			BF_{10}	0.28	0.26	0.21	0.21	0.26	0.20
AG	left	3	*n*	35	25	35	35	35	35
			r	-.032	-.024	-.115	-.173	-.094	-.103
			BF_{10}	0.21	0.25	0.26	0.34	0.24	0.25
	right	11	*n*	39	29	39	39	39	39
			r	-.020	.031	-.309	-.097	-.143	-.076
			BF_{10}	0.20	0.23	1.17	0.24	0.29	0.22
SMG	left	1	*n*	38	29	38	38	38	38
			r	.161	-.086	-.069	-.153	-.067	-.220
			BF_{10}	0.32	0.25	0.22	0.30	0.22	0.47
	right	9	*n*	39	29	39	39	39	39
			r	.212	-.074	-.209	.168	-.133	.052
			BF_{10}	0.45	0.25	0.44	0.33	0.27	0.21

Notes. BF_{10} is given for Bayesian correlations of carry/borrow effects in behavior (ACC – accuracy, RT – reaction time) and brain activation (per ch – channel, based on final GLM with HRF peak at 9 s), with $BF_{10} > 3$ indicating evidence for an effect (marked in **bold**).

Abbreviations of brain regions: IFG – inferior frontal gyrus, MFG – middle frontal gyrus, IPS –intraparietal sulcus, SMG – supramarginal gyrus, AG – angular gyrus.

Table I. *Brain-behavior-correlations of the carry effect in younger adults.*

variable / brain region		ch		processing speed (score)	working memory (ACC)	verbal intelligence (score)	non-verbal intelligence (score)	carry effect (ACC)	carry effect (RT)
ACC			*n*	36	56	59	59		
			r	-.018	.168	.054	.074		
			BF_{10}	0.21	0.35	0.18	0.19		
RT			*n*	36	**56**	59	59		
			r	-.286	**-.334**	-.262	-.182		
			BF_{10}	0.82	**3.68**	1.15	0.41		
IFG	left	5	*n*	36	55	58	58	58	58
			r	.110	-.063	-.138	.093	.188	.293
			BF_{10}	0.25	0.19	0.28	0.21	0.43	1.87
		8	*n*	35	53	56	56	56	56
			r	.083	-.013	.029	.076	-.096	.229
			BF_{10}	0.24	0.17	0.17	0.19	0.21	0.68
	right	13	*n*	36	55	58	58	58	58
			r	.123	-.036	-.106	-.034	.109	.219
			BF_{10}	0.27	0.17	0.22	0.17	0.23	0.62
		16	*n*	36	55	58	58	58	58
			r	.257	.022	-.123	-.131	-.029	-.039
			BF_{10}	0.62	0.17	0.25	0.26	0.17	0.17
MFG	left	4	*n*	36	54	57	57	57	57
			r	.011	.009	-.023	.049	.069	.113
			BF_{10}	0.21	0.17	0.17	0.18	0.19	0.23
		6	*n*	36	55	58	58	58	58
			r	-.076	-.120	-.177	-.121	.052	.273
			BF_{10}	0.23	0.24	0.39	0.25	0.18	1.33
		7	*n*	36	55	58	58	58	58
			r	.150	-.038	-.113	-.085	.130	.287
			BF_{10}	0.30	0.18	0.23	0.20	0.26	1.67
	right	12	*n*	36	55	58	58	58	58
			r	.120	-.067	.004	.008	.187	.107
			BF_{10}	0.26	0.19	0.16	0.16	0.43	0.22
		14	*n*	36	55	58	58	58	58
			r	-.011	-.130	-.082	-.124	.036	.214
			BF_{10}	0.21	0.26	0.20	0.25	0.17	0.58
		15	*n*	36	55	58	58	58	**58**
			r	.092	-.102	-.203	-.043	.035	**.325**
			BF_{10}	0.24	0.22	0.52	0.17	0.17	**3.34**
IPS	left	2	*n*	36	54	57	57	57	**57**
			r	-.156	-.152	-.027	-.074	.158	**.348**
			BF_{10}	0.31	0.31	0.17	0.19	0.33	**5.16**
	right	10	*n*	36	55	58	58	58	58
			r	-.138	-.163	-.126	-.117	.246	.298
			BF_{10}	0.28	0.34	0.25	0.24	0.88	2.03
AG	left	3	*n*	35	53	56	56	56	56
			r	.128	-.002	-.100	-.139	.171	.282
			BF_{10}	0.27	0.17	0.22	0.28	0.36	1.44
	right	11	*n*	36	55	58	58	58	58
			r	.014	-.179	-.083	-.034	.084	.260
			BF_{10}	0.21	0.39	0.20	0.17	0.20	1.08
SMG	left	1	*n*	35	**53**	56	56	56	56
			r	.171	**-.346**	-.020	-.072	.031	.302
			BF_{10}	0.33	**3.94**	0.17	0.19	0.17	2.01
	right	9	*n*	35	53	56	56	56	56
			r	.192	-.119	-.090	.002	.016	.272
			BF_{10}	0.38	0.24	0.21	0.17	0.17	1.23

Notes. BF_{10} is given for Bayesian correlations of carry/borrow effects in behavior (ACC – accuracy, RT – reaction time) and brain activation (per ch – channel, based on final GLM with HRF peak at 9 s), with $BF_{10} > 3$ indicating evidence for an effect (marked in **bold**).

Abbreviations of brain regions: IFG – inferior frontal gyrus, MFG – middle frontal gyrus, IPS –intraparietal sulcus, SMG – supramarginal gyrus, AG – angular gyrus.

Table J. *Brain-behavior-correlations of the borrow effect in younger adults.*

variable / brain region		ch		processing speed (score)	working memory (ACC)	verbal intelligence (score)	non-verbal intelligence (score)	borrow effect (ACC)	borrow effect (RT)
ACC			*n*	36	56	**59**	59		
			r	.155	.050	**.319**	.035		
			BF_{10}	0.31	0.18	**3.10**	0.17		
RT			*n*	36	**56**	**59**	59		
			r	-.076	**-.347**	**-.459**	-.194		
			BF_{10}	0.23	**4.78**	**> 100**	0.47		
IFG	left	5	*n*	36	55	58	58	58	58
			r	-.108	.157	-.103	-.011	-.100	.216
			BF_{10}	0.25	0.32	0.22	0.16	0.22	0.60
		8	*n*	35	**53**	56	56	56	**56**
			r	-.133	**-.342**	-.277	-.059	-.197	**.484**
			BF_{10}	0.28	**3.63**	1.33	0.18	0.47	**> 100**
	right	13	*n*	36	55	58	58	58	58
			r	-.153	.106	-.024	.033	-.056	.014
			BF_{10}	0.30	0.23	0.17	0.17	0.18	0.17
		16	*n*	36	55	58	58	58	58
			r	.103	.112	.033	-.076	.136	-.062
			BF_{10}	0.25	0.23	0.17	0.19	0.27	0.18
MFG	left	4	*n*	36	54	57	57	57	**57**
			r	.077	-.032	-.089	.035	.037	**.357**
			BF_{10}	0.23	0.17	0.20	0.17	0.17	**6.20**
		6	*n*	36	55	58	58	58	**58**
			r	-.065	-.041	-.118	.018	-.101	**.446**
			BF_{10}	0.22	0.18	0.24	0.17	0.22	**65.93**
		7	*n*	36	55	58	58	58	58
			r	-.038	.142	.101	-.031	.184	.107
			BF_{10}	0.21	0.28	0.22	0.17	0.42	0.22
	right	12	*n*	36	55	58	58	58	58
			r	.256	.205	.178	.132	.175	-.089
			BF_{10}	0.62	0.51	0.39	0.26	0.38	0.20
		14	*n*	36	55	58	58	58	58
			r	-.198	.005	.002	-.114	.003	.077
			BF_{10}	0.40	0.17	0.16	0.23	0.16	0.19
		15	*n*	36	55	58	58	58	58
			r	-.190	.052	-.059	-.023	-.090	.149
			BF_{10}	0.38	0.18	0.18	0.17	0.20	0.30
IPS	left	2	*n*	36	54	57	57	57	57
			r	-.219	.113	.079	.069	.224	-.073
			BF_{10}	0.46	0.23	0.20	0.19	0.65	0.19
	right	10	*n*	36	55	58	58	58	58
			r	-.324	-.012	-.003	.057	.077	.064
			BF_{10}	1.23	0.17	0.16	0.18	0.19	0.18
AG	left	3	*n*	35	53	56	56	56	56
			r	-.070	-.018	.071	.122	.100	.180
			BF_{10}	0.23	0.17	0.19	0.25	0.22	0.39
	right	11	*n*	36	55	58	58	58	58
			r	-.030	-.192	-.012	-.257	.018	.152
			BF_{10}	0.21	0.44	0.16	1.04	0.17	0.31
SMG	left	1	*n*	35	53	56	56	56	56
			r	-.314	.033	-.001	-.040	-.031	.127
			BF_{10}	1.07	0.18	0.17	0.17	0.17	0.26
	right	9	*n*	35	53	56	56	56	56
			r	-.037	-.033	-.125	.001	-.038	.228
			BF_{10}	0.22	0.18	0.25	0.17	0.17	0.67

Notes. BF_{10} is given for Bayesian correlations of carry/borrow effects in behavior (ACC – accuracy, RT – reaction time) and brain activation (per ch – channel, based on final GLM with HRF peak at 9 s), with $BF_{10} > 3$ indicating evidence for an effect (marked in **bold**).

Abbreviations of brain regions: IFG – inferior frontal gyrus, MFG – middle frontal gyrus, IPS –intraparietal sulcus, SMG – supramarginal gyrus, AG – angular gyrus.

Table K. *Brain-behavior-correlations of the carry effect in older adults.*

variable / brain region		ch		processing speed (score)	working memory (ACC)	verbal intelligence (score)	non-verbal intelligence (score)	carry effect (ACC)	carry effect (RT)
ACC			*n*	**48**	46	51	51		
			r	**.386**	.297	.264	.175		
			BF_{10}	**6.41**	1.29	0.96	0.36		
RT			*n*	48	46	51	51		
			r	-.207	-.090	-.124	-.114		
			BF_{10}	0.47	0.22	0.25	0.24		
IFG	left	5	*n*	45	44	48	48	49	49
			r	.160	-.019	-.038	.107	.082	.135
			BF_{10}	0.32	0.19	0.19	0.23	0.21	0.27
		8	*n*	46	44	49	49	50	50
			r	-.319	-.259	-.214	.163	-.093	.194
			BF_{10}	1.78	0.76	0.51	0.33	0.22	0.43
	right	13	*n*	46	44	49	49	49	49
			r	-.077	.005	-.039	.215	-.047	.162
			BF_{10}	0.21	0.19	0.18	0.52	0.19	0.32
		16	*n*	45	43	48	48	49	49
			r	-.090	-.010	-.108	.213	.097	.139
			BF_{10}	0.22	0.19	0.23	0.50	0.22	0.28
MFG	left	4	*n*	45	43	48	48	49	49
			r	.108	.098	.041	.007	-.027	.139
			BF_{10}	0.24	0.23	0.19	0.18	0.18	0.28
		6	*n*	44	42	47	47	48	**48**
			r	-.016	-.170	-.092	-.009	-.026	**.399**
			BF_{10}	0.19	0.34	0.22	0.18	0.18	**8.25**
		7	*n*	43	42	46	46	47	47
			r	.253	.211	.206	-.021	.086	.138
			BF_{10}	0.69	0.46	0.46	0.19	0.21	0.28
	right	12	*n*	46	44	49	49	49	**49**
			r	-.133	.119	.023	.173	-.130	**.356**
			BF_{10}	0.27	0.25	0.18	0.35	0.26	**3.78**
		14	*n*	46	44	49	49	50	50
			r	.106	-.024	.011	.072	.002	.290
			BF_{10}	0.23	0.19	0.18	0.20	0.18	1.34
		15	*n*	45	43	48	48	49	49
			r	.201	.056	.013	.049	.066	.152
			BF_{10}	0.44	0.20	0.18	0.19	0.20	0.30
IPS	left	2	*n*	44	42	47	47	48	48
			r	-.022	-.055	-.065	-.021	-.152	.202
			BF_{10}	0.19	0.20	0.20	0.18	0.30	0.45
	right	10	*n*	45	43	48	48	49	49
			r	.143	.048	.033	.145	-.114	.135
			BF_{10}	0.28	0.20	0.18	0.29	0.24	0.27
AG	left	3	*n*	43	42	46	46	47	47
			r	.248	-.074	.003	.129	.056	.105
			BF_{10}	0.66	0.21	0.18	0.26	0.19	0.23
	right	11	*n*	46	44	49	49	50	50
			r	.304	-.056	.048	.131	-.052	-.043
			BF_{10}	1.41	0.20	0.19	0.26	0.19	0.18
SMG	left	1	*n*	45	43	48	48	49	49
			r	.143	.074	.076	.086	-.052	.036
			BF_{10}	0.28	0.21	0.20	0.21	0.19	0.18
	right	9	*n*	46	44	49	49	50	50
			r	.167	.076	.103	.042	.013	-92.410
			BF_{10}	0.34	0.21	0.23	0.19	0.18	0.18

Notes. BF_{10} is given for Bayesian correlations of carry/borrow effects in behavior (ACC – accuracy, RT – reaction time) and brain activation (per ch – channel, based on final GLM with HRF peak at 9 s), with $BF_{10} > 3$ indicating evidence for an effect (marked in **bold**).

Abbreviations of brain regions: IFG – inferior frontal gyrus, MFG – middle frontal gyrus, IPS –intraparietal sulcus, SMG – supramarginal gyrus, AG – angular gyrus.

Table L. *Brain-behavior-correlations of the borrow effect in older adults.*

variable / brain region		ch		processing speed (score)	working memory (ACC)	verbal intelligence (score)	non-verbal intelligence (score)	borrow effect (ACC)	borrow effect (RT)
ACC			*n*	48	46	51	**51**		
			r	-.101	-.041	.074	**.373**		
			BF_{10}	0.23	0.19	0.20	**5.95**		
RT			*n*	48	46	51	51		
			r	-.124	-.127	-.148	.002		
			BF_{10}	0.25	0.26	0.29	0.18		
IFG	left	5	*n*	45	44	48	48	49	49
			r	.131	-.026	.007	-.093	-.185	-.126
			BF_{10}	0.27	0.19	0.18	0.22	0.39	0.26
		8	*n*	46	44	49	49	50	50
			r	-.042	-.159	-.049	.233	-.024	.260
			BF_{10}	0.19	0.31	0.19	0.63	0.18	0.89
	right	13	*n*	46	44	49	49	49	49
			r	.119	.200	-.032	.129	.117	.012
			BF_{10}	0.25	0.43	0.18	0.26	0.24	0.18
		16	*n*	45	43	48	48	49	49
			r	.163	.108	-.059	.262	.133	.009
			BF_{10}	0.32	0.24	0.20	0.86	0.27	0.18
MFG	left	4	*n*	45	43	48	48	49	49
			r	.244	.051	.025	-.062	.126	-.186
			BF_{10}	0.66	0.20	0.18	0.20	0.26	0.39
		6	*n*	44	42	47	47	48	48
			r	-.066	-.112	-.078	-.133	.046	-.035
			BF_{10}	0.21	0.24	0.21	0.27	0.19	0.19
		7	*n*	**43**	42	46	46	47	47
			r	**.386**	.233	.146	.072	-.055	.087
			BF_{10}	**4.46**	0.56	0.29	0.21	0.19	0.21
	right	12	*n*	46	44	49	49	49	49
			r	.033	-.040	-.030	.144	.257	-.022
			BF_{10}	0.19	0.19	0.18	0.29	0.83	0.18
		14	*n*	46	44	49	49	50	50
			r	.113	.051	-.096	.150	.193	.131
			BF_{10}	0.24	0.20	0.22	0.30	0.43	0.26
		15	*n*	45	43	48	48	49	49
			r	.184	.031	-.005	.097	.057	.042
			BF_{10}	0.38	0.19	0.18	0.22	0.19	0.19
IPS	left	2	*n*	44	42	47	47	48	48
			r	.248	.072	.116	.206	.092	.108
			BF_{10}	0.67	0.21	0.24	0.46	0.22	0.23
	right	10	*n*	45	43	48	**48**	49	49
			r	.210	.022	.129	**.381**	.194	.022
			BF_{10}	0.47	0.19	0.26	**5.78**	0.42	0.18
AG	left	3	*n*	43	42	46	46	47	47
			r	.267	.082	.030	.179	.068	-.155
			BF_{10}	0.81	0.22	0.19	0.37	0.20	0.31
	right	11	*n*	**46**	44	49	49	50	50
			r	**.365**	.248	.144	.283	.127	-.069
			BF_{10}	**3.77**	0.67	0.29	1.17	0.26	0.20
SMG	left	1	*n*	45	43	48	48	49	49
			r	.262	.241	.113	.145	-.018	-.026
			BF_{10}	0.80	0.62	0.24	0.29	0.18	0.18
	right	9	*n*	46	44	49	49	50	50
			r	.340	.114	.065	.138	.032	-.016
			BF_{10}	2.44	0.25	0.20	0.28	0.18	0.18

Notes. BF_{10} is given for Bayesian correlations of carry/borrow effects in behavior (ACC – accuracy, RT – reaction time) and brain activation (per ch – channel, based on final GLM with HRF peak at 9 s), with $BF_{10} > 3$ indicating evidence for an effect (marked in **bold**).

Abbreviations of brain regions: IFG – inferior frontal gyrus, MFG – middle frontal gyrus, IPS –intraparietal sulcus, SMG – supramarginal gyrus, AG – angular gyrus.
