# Supplementary material for: No developmental fronto-parietal shift in brain activation during mental arithmetic across the lifespan: A registered report
Source: PLoS One. 2026 Aug 5;21(8):e0353139. doi: 10.1371/journal.pone.0353139 (PMC13440826; doi:10.1371/journal.pone.0353139)
Supplement: Supplementary file 2 [file pone.0353139.s003.pdf]

Dear Editor,

Thank you for the opportunity to revise our manuscript and respond to the reviewer comments on our Registered Report Stage II submission. We appreciate the reviewers' careful reading of the manuscript and their constructive feedback.

However, we would like to respectfully note that the majority of the comments concern the framing and relevance of the study, the introduction and hypotheses, and some methodological and design-related aspects of the study. As this manuscript is currently under review as a Registered Report Stage II submission, these elements were already evaluated, approved, and formally accepted during the Stage I review process. In the Registered Report format, the primary purpose of Stage I peer review is to assess and approve the study rationale, hypotheses, methodology, and analysis plan prior to data collection. Consequently, substantial modifications to the introduction or methods are generally not possible at Stage II, unless specifically requested by the editor.

Accordingly, we addressed these comments in two ways: First, whereever possible, we implemented minor revisions in the introduction for purposes of clarity, precision, and readability – without changing the content. Second, we acknowledged and discussed the reviewers' concerns in the discussion and limitations sections. As substantive revisions to the theoretical framing, hypotheses, or methodological choices would contradict the principles and procedural structure of Registered Reports, we did not implement them at this stage - unless the editor explicitly requires such changes.

A point-by-point response to all reviewer comments follows below. The manuscript has been revised accordingly, and all changes are marked in yellow.

As requested, here is the statement about the funders: "The funders had no role in study design, data collection and analysis, decision to publish, or preparation of the manuscript."

Thank you for your time and consideration.

Sincerely,
Christina Artemenko

Reviewer #1: This is an interesting and overall well-written manuscript. The study examines arithmetic processing across the lifespan by comparing elementary school children, younger adults, and older adults, with the goal of characterizing behavioral and neural correlates of arithmetic at different stages: during initial learning, after consolidation, and later in aging. The focus on two-digit addition and subtraction problems with and without carrying/borrowing is potentially valuable. At the same time, several aspects of the theoretical framing, hypothesis formulation, and interpretation require clarification and greater precision. I encourage the authors to strengthen the conceptual rationale for the study design, more clearly justify how the chosen task can test the proposed hypotheses, and moderate some of the claims in the discussion.

Reply: We would like to thank the reviewer for the evaluation of our manuscript.

Major comments

1. The rationale for the study should not rely on novelty alone

On p. 5, the manuscript states that because neuroimaging research on arithmetic in older adults has not been conducted before, the project opens a new research field in numerical cognition. I do not find this argument sufficiently convincing on its own. The importance of the study should be grounded primarily in its theoretical contribution and in the extent to which the design can adjudicate between competing predictions. I recommend revising this section to explain more clearly why studying this shift including older adults is relevant, rather than emphasizing that the topic has not previously been studied.

Reply: The rationale of this study is to replicate the developmental shift, to fill the gap and extend it to aging, and to uncover the processes underlying behavior that change during development. Concerning aging, it is not only about novelty, but also about the underlying mechanisms, as a frontal decrease (as in childhood development) might point at a further functional specialization, while a frontal increase might point at compensatory mechanisms during aging.

In general, the study addresses theoretical relevance, by testing and extending developmental models of numerical cognition to the entire lifespan (p. 4-5 and p. 8). Regarding practical relevance, the carry and borrow operations are challenges to learn during math education (p. 4 and additionally added to the discussion on p. 28), and such operations on two-digit numbers are highly relevant for daily life up to old age (e.g., shopping, p. 5). Therefore, this study contributes to the understanding of how arithmetic skills change across the lifespan and how individuals of different ages process arithmetic difficulty.

While these points are already incorporated at various places throughout the introduction, additional substantial modifications to the introduction are not possible at this stage of the Registered Report (including theoretical framing and rationale).

2. The framing of single-digit versus two-digit arithmetic needs to be reconsidered

The paragraph on p. 5 about the limitations of prior work focusing on single-digit arithmetic is currently not fully convincing. I would avoid characterizing that literature as "unfortunate." In fact, single-digit arithmetic is highly relevant in elementary school and has been central to prior developmental work on arithmetic-related brain activation. More importantly, the manuscript should engage more directly with the implications of using two-digit addition and subtraction rather than single-digit arithmetic. This is especially critical because the fronto-parietal shift literature invoked by the authors appears to derive largely from studies using single-digit operations (e.g. Rivera et al., 2005). Two-digit arithmetic may

recruit additional procedural, working-memory, and place-value-related processes, which may reduce comparability with that earlier literature. As a result, it is not yet clear whether the present design is ideally suited to test predictions derived from prior evidence on the developmental fronto-parietal shift. I therefore recommend that the authors revise this section to justify more explicitly the use of two-digit arithmetic for the present research questions, acknowledge more clearly how this choice differs from the literature on which the hypotheses are based, and discuss whether the use of two-digit operations may itself contribute to the absence of evidence for the expected shift.

Reply: This is a very important comment. When having a closer look at the studies that fully or at least partially support the fronto-parietal shift hypothesis, most of them use single-digit number comparison (Ansari et al., 2005) or single-digit arithmetic (addition and subtraction in Rivera et al., 2005; addition in Kucian et al., 2008); subtraction in Chang et al., 2016) and only one study uses two-digit addition and subtraction (Artemenko et al., 2018); for further studies see the review by Peters & De Smedt (2017). Therefore, the current neuroimaging study is important, as the fronto-parietal shift hypothesis was rarely tested for multi-digit arithmetic before.

In response, we first implemented minor revisions in the introduction to improve clarity and readability. Namely, we removed the term "unfortunately" (p. 5). However, due to the Registered Report format, no additional substantial changes were considered for the introduction at this stage. Instead, we now discuss in the discussion section that previous evidence was primarily based on single-digit arithmetic and may therefore not necessarily generalize to two-digit arithmetic processing.

First, we have revised the beginning of the discussion to prominently frame this interpretation as a theoretical insight instead of describing it as a surprising null result (p. 26 f.): "This suggests that solving complex two-digit arithmetic requires procedural calculation steps and place-value computation, being subserved by the fronto-parietal network independent of age. Due to the high task demands, age-related changes in brain activation – such as the gradual developmental shift from frontal to parietal activation or general developmental changes – were not observed, that might be limited to single-digit arithmetic. Nevertheless, place-value computation, as indicated by the carry and borrow effects, became more efficient across the whole lifespan."

Second, we revised the interpretation of the frontal findings by referring to the "procedural strategies" (p. 26) and the increased task demands of two-digit arithmetic (p. 26): "Accordingly, no qualitative differences in brain activation patterns were observed, and children relied on the same frontal brain resources as adults, reflecting the high task demands of two-digit arithmetic that might have masked age-related differences."

Third, we revised the interpretation of the IPS findings (p. 27): "Hence the functional specialization of the IPS during development does not generalize from single-digit basic number magnitude processing [80,83–85] to more complex arithmetic involving multi-digit numbers. Thus, the role of the IPS in arithmetic might depend more on the operations and complexity involved than on age [87,88], or it may emerge earlier in development, prior to grade 3, rather than in parallel with the decrease in frontal activation [82]. Taken together, evidence for a developmental fronto-parietal shift in brain activation during two-digit arithmetic is limited and, if present at all, only partially supported for frontal activation decrease when comparing children and adults."

Furthermore, we clarified the interpretation of our results in relation to the literature: "Consequently, the developmental fronto-parietal shift might not reflect a shift toward functional specialization in the IPS, but rather increasing automatization in the AG and SMG associated with changes in strategy use from procedural calculation to fact retrieval [89,90]. This might explain why the developmental fronto-parietal shift only applies to single-digit arithmetic, which

increasingly relies on fact retrieval across the lifespan, but not to two-digit arithmetic, which mostly relies on procedural strategies [91,92]."

Finally, we revised the abstract accordingly (p. 3): "Thus, the developmental fronto-parietal shift in arithmetic processing might only apply to single-digit arithmetic – but not to two-digit arithmetic across the lifespan."

3. The concept of a fronto-parietal shift "continuing during aging" needs clarification

The manuscript repeatedly refers to whether the developmental fronto-parietal shift "continues during aging." I found this formulation conceptually unclear. A shift typically refers to a change from one state or pattern to another. Describing such a shift as "continuing" during aging is difficult to interpret unless the authors mean that the shift is gradual and extends across additional stages of the lifespan. The authors should define more precisely what they mean by this idea. For example, do they predict a continued decrease in frontal reliance and increase in parietal reliance beyond young adulthood? A maintenance of the adult pattern into older age? This issue should be clarified both in the Introduction and in the Discussion.

Reply: Indeed the shift is meant to be gradual. Decreases in frontal activation and increases in parietal activation are meant be located in the fronto-parietal network which is active during arithmetic – only to a different degree depending on the brain region. We clarified this by adding "gradual" to the fronto-parietal shift in the abstract (p. 3) and introduction (p. 4, 5, 8). The hypothesis on a continuation of the gradual shift during aging captures the proposed further changes during aging and does not need any revisions: "if the developmental fronto-parietal shift generalizes to the whole lifespan, older adults might show less frontal activation (left IFG and bilateral MFG) and increased parietal activation (left IPS) in comparison to younger adults" (p. 8).

While the revisions in the introduction needed to be minimal, we revised the discussion more thoroughly. We use the term "gradual fronto-parietal shift" in the discussion (p. 26-27) and explicitly reported which evidence argues for or against the shift in the conclusions (p. 30): "Therefore, the present findings suggest that the developmental fronto-parietal shift may not generalize from single-digit to two-digit arithmetic and arithmetic effects. While there is some evidence for a decrease in frontal activation from childhood to adulthood, no corresponding increase in parietal activation was observed, and no comparable age-related changes were evident in adulthood."

4. The link between carrying/borrowing and the fronto-parietal shift requires a stronger rationale

On p. 7, the manuscript suggests that carry and borrow effects are associated with increased frontal and parietal activation, and appears to use this as part of the rationale for including these manipulations. However, it is not yet clear why these manipulations are necessary for testing hypotheses about the fronto-parietal shift. The authors should explain more explicitly: 1) why carry and borrow were included. 2) whether these manipulations are central to the main hypotheses or secondary to them. 3) how complexity-related effects are expected to inform the broader question of age-related changes in arithmetic processing. In the current version of the manuscript, the connection between arithmetic complexity effects and the proposed lifespan fronto-parietal account remains underdeveloped.

Reply: The carry and borrow operations reflect the highest level of place-value processing (computations across place-values, p. 5) and are represented in the frontal-parietal network. While task-based approaches (comparing brain activation during arithmetic tasks between

different age groups) are limited, as activation patterns might reflect general processes that are not specific to the task at-hand, effect-based approaches (comparing differences in brain activation between complex and simple arithmetic tasks between different age groups) subtract such general processes and thus allow for evaluating more specific task-relevant processes – in this case place-value computation.

To differentiate between the different approaches, we introduced the hypotheses on all three levels: H1 following the task-based approach, H2 the effect-based approach, and H3 the effect-based approach specific on the underlying processing characteristic (p. 8). Therefore, the hypotheses covered all approaches including task performance, carry/borrow effects, and the underlying processes. To respond to the reviewer's questions: (1) Carry and borrow were included as they specifically assess place-value manipulation and are represented in the fronto-parietal network of arithmetic processing. (2) The carry and borrow effects were central to H2 and H3 but not to H1. (3) As these effects particularly target task-relevant processes (place-value computation), they might inform developmental changes particularly to domain-specific processes when comparing with general changes.

As the introduction can not substantially changed at this stage, we stated the results concerning arithmetic performance and arithmetic effects in relation to the developmental shift separately in the conclusions (p. 30), in order to resample the structure of the discussion.

5. Some hypotheses are difficult to interpret and need to be reformulated

Several hypotheses would benefit from greater precision.

On p. 8, the hypothesis that children should show increased frontal activation and less parietal activation than younger adults is presented as following from previous work on the developmental fronto-parietal shift. However, because that literature is based primarily on single-digit arithmetic, the authors should acknowledge that applying the same prediction to two-digit arithmetic requires an additional assumption.

Reply: Please see our response to comment 2. We added the argument about single- vs. two-digit arithmetic processing in the discussion (not in the introduction).

Also on p. 8, the paragraph discussing older adults is difficult to follow because it combines distinct possibilities into a single sentence. I suggest separating the alternatives more clearly. For instance, one possibility is that arithmetic processing is relatively preserved in aging, which would predict a pattern similar to that of younger adults. A different possibility is that older adults rely more strongly on compensatory mechanisms, which may predict greater frontal recruitment. These alternatives should be presented distinctly.

Reply: We revised this part accordingly (p. 8): "however, if arithmetic is not affected by aging, activation might be similar in older and younger adults; finally, if arithmetic is affected by aging and needs compensation, older adults might show increased frontal activation in comparison to younger adults." Nevertheless, if these changes to the hypothesis are considered too substantial for a Registered Report Stage II submission, we are of course willing to revert them.

Likewise, the section on the predicted developmental trajectory of carry and borrow effects is currently confusing. The manuscript proposes alternative patterns, but it is not sufficiently clear what neural

changes are expected in each case, or how the data would distinguish between them. This should be rewritten more explicitly.

Reply: We revised the part of the hypothesis accordingly (p. 8): “At the neural level, the continuous carry and borrow effects (unit sum and unit difference) should rely on parietal activation (left IPS), while the categorical carry and borrow effects mainly rely on frontal activation (left IFG and bilateral MFG) across age groups.” Again, if these changes are too substantial, we will revert them.

The differentiation on the neural level would be given according to the first part of the hypothesis, stating a categorical effect in children (meaning frontal activation for the categorical effect in children), both categorical and continuous in younger adults, and continuous in older adults (meaning parietal activation correlated to unit sum/difference in older adults). As robust results were only present at the behavioral level, the neural level is not further discussed in the discussion.

6. The discussion should interpret the null evidence more cautiously

My main concern in the Discussion is that several conclusions are stated too strongly relative to the design and method. The authors do note that the use of fNIRS may have limited the ability to detect a fronto-parietal shift, which is appropriate. However, I think the discussion should also acknowledge additional reasons why such evidence may not have been observed in the present study. In particular, the authors should discuss more fully the use of two-digit rather than single-digit arithmetic. Because two-digit addition and subtraction can recruit a mixture of strategies and impose broader cognitive demands, this paradigm may not be optimal for isolating a developmental shift toward parietal specialization. Relatedly, if the task maintains procedural demands at a relatively high level for all groups, age differences in frontal recruitment may be attenuated or masked. I suggest that authors are more clear about the possibility that task complexity may have sustained frontal control demands across all age groups, thereby making developmental differences harder to detect.

For these reasons, statements such as “our results challenge the existence of the fronto-parietal shift” seem too strong. A more appropriate conclusion would be something along the following lines: “We did not observe evidence for the fronto-parietal shift in this sample using this fNIRS paradigm with two-digit addition and subtraction.” That wording is more consistent with the actual design and with the limits of the inferences that can be drawn.

Reply: In response to this comment, we revised the discussion including limitations and conclusions. We refer now also specifically to frontal and parietal cortical activation that was covered by fNIRS in the current study. Here are three examples of revisions:

In the beginning of the discussion (p. 26): “This suggests that solving complex two-digit arithmetic requires procedural calculation steps and place-value computation, being subserved by the fronto-parietal network independent of age. Due to the high task demands, age-related changes in brain activation – such as the gradual developmental shift from frontal to parietal activation or general developmental changes – were not observed, that might be limited to single-digit arithmetic. Nevertheless, place-value computation, as indicated by the carry and borrow effects, became more efficient across the whole lifespan.”

In the discussion on the frontal results (p. 28): “This might be explained by the increased frontal control demands associated with procedural calculation strategies across all age groups [96], which might obscure smaller age-related differences.”

In the conclusions: “Based on this first neuroimaging study investigating arithmetic development across the lifespan, we conclude that processing two-digit arithmetic is remarkably stable within the frontal and parietal cortex.” (p. 30)

7. Some claims about IPS activation are overstated

On p. 26, the manuscript states that the stability of IPS activation during aging points to functional specialization for number magnitude processing that is maintained throughout adulthood without deterioration. I think this statement goes beyond what the present data can support. Reliance on IPS during addition and subtraction does not, by itself, permit strong conclusions about number magnitude refinement. I recommend revising this passage more cautiously and tying the interpretation more directly to the specific task demands used here.

Reply: We agree that our task does not directly assess number magnitude processing and revised the interpretation of the IPS findings accordingly (p. 26): “Hence the functional specialization of the IPS during development does not generalize from single-digit basic number magnitude processing [80,83–85] to more complex arithmetic involving multi-digit numbers. Thus, the role of the IPS in arithmetic might depend more on the operations and complexity involved than on age [...].” And also later (p. 27): “By contrast, the stability of IPS activation during aging points to a functional specialization for number processing and procedural calculation strategies [74,89,96]”.

Minor comments

• p. 4, line 84: I suggest revising the sentence to: “Moreover, the question arises as to how these developmental changes progress during aging.”

Reply: We revised it as suggested.

• p. 5, line 94: This paragraph would benefit from rewriting for clarity and balance. In particular, I would avoid implying that the focus on single-digit arithmetic in prior work is inherently problematic.

Reply: Please see our response to comment 2.

• p. 8, line 195: Please provide a clearer rationale for the prediction that carry and borrow effects should be categorical in children, mixed in younger adults, and continuous in older adults.

Reply: The rationale for this hypothesis comes from the following text (p. 6): “Furthermore, the underlying processing characteristics of these effects might change, since the carry and borrow effects seem to be categorical effects in elementary school children [27], while the carry effect in younger adults relies on continuous processing characteristics as well, as assessed by the unit sum [26,31,36]. This suggests that primarily domain-general processes are driving the carry and borrow effects in children, while in younger adults, domain-specific processes are additionally driving the carry effect. Considering the general cognitive decline during aging, the carry and borrow effects might be mainly driven by domain-specific processes in older adults, but empirical evidence is still missing.” At this stage we refrain from substantial changes in the hypotheses.

• p. 22, Table 2: Please define all abbreviations in the table note, including “ch.” Also, centering the contents may improve readability.

Reply: We added the abbreviation for channel in the notes of the table and centered “ch” in the header (see Table 2).

• p. 24, Figures 3 and 4: These figures are currently very difficult to read. I recommend separating frontal and posterior results into different panels or figures and making significant effects easier to identify visually.

> Reply: We revised the figures by increasing font size, adding stars for supported effects, and adding figure captions including the presented effects and the abbreviations (see Figures 3 and 4).

Overall, I think this manuscript addresses an interesting question and has the potential to make a valuable contribution. To strengthen the paper, I encourage the authors to sharpen the theoretical rationale, clarify the hypotheses, and adopt a more cautious interpretation of the findings. In particular, the manuscript would benefit from a more explicit discussion of how the use of two-digit arithmetic and fNIRS affects comparability with prior literature on the developmental fronto-parietal shift.

> Reply: We thank the reviewer for all the constructive comments. By implementing them, the theoretical contribution of our study increased and limitations were explicitly mentioned.

Reviewer #2: The manuscript examines addition and subtraction problem solving in elementary school students, younger adults, and older adults using fNIRS. The authors hypothesized a fronto-parietal shift during arithmetic problem solving across age and the complexity (carrying/borrowing) of the problems should result in enhanced activity in frontal areas, especially in children. The main findings did not support the hypotheses: no fronto-parietal shift was found across age groups and the level of enhancement due to complexity in the frontal area was smaller in children than in adults in right MFG. Overall, I find myself confused by the results and not fully convinced by the authors' interpretation. The theoretical setup of the manuscript is also a bit unclear. I will explain my points in detail below.

Reply: We thank the reviewer for the thoughtful evaluation of our study. We addressed the comments below and particularly revised the conclusions to clearly state our findings.

Major concerns:

1. Methodologically I'm concerned whether fNIRS is the best tool to investigate this research question and whether the authors picked the right target regions in the frontal cortex. Arithmetic problem solving involves distributed brain regions, mainly including ventral visual pathway, language system, medial temporal memory system, parietal cortex, and frontal cognitive control system. Only examining the lateral frontal cortex and parietal cortex could result in findings hard to interpret. Moreover, the authors highlighted the relationship between frontal cortex and working memory. I'm worried that the authors might have missed the important dorsomedial prefrontal cortex/dorsal ACC and insula (salience network) as these regions are well known for their domain general functions and have been frequently found during arithmetic problem solving (see Sokolowski, Hawes, & Ansari, 2022 and Unal, Park, Simsek, Menon, & Geary, 2025 meta-analysis).

Reply: We thank the reviewer for this comment. We agree that arithmetic processing relies on a widespread brain network and that not all relevant regions were covered by the fNIRS probeset used in the present study. The references to the meta-analyses mentioned by the reviewer were published after the Registered Report stage I had been accepted; we therefore appreciate the opportunity to incorporate these findings into the revised discussion. In response to the comment, we added the references where appropriate and revised overstated claims throughout the discussion (p. 27, 28).

We further expanded the limitations section to more explicitly acknowledge the restricted spatial coverage of fNIRS (p. 30): "Furthermore, due to its limited spatial and depth resolution, fNIRS is restricted to cortical regions and cannot capture activation in subcortical regions or fine-grained local differences within cortical regions. In addition, arithmetic processing relies on a distributed network that includes regions such as the dorsomedial prefrontal cortex, the cingulate gyrus, and the insula [74,89,96]. Since our fNIRS probeset only covered lateral frontal and parietal areas, age-related differences might still occur in other brain regions."

2. I'm having a hard time understanding the rationale for the exploratory analysis on the mean amplitude and peak latency. I'm not an expert on fNIRS but more on fMRI. Conceptually this exploratory analysis is analyzing the amplitude and peak latency of blood oxygen level change. Indeed, the exploratory findings support the hypotheses better than the GLM findings. However, many of the exploratory results contradict with the GLM results. What does that mean? Which analysis should we trust and why? The authors need to provide stronger rationale for the exploratory analysis.

Reply: We agree that the complexity of analyses requires further explanations. The analysis of fNIRS data is not as standardized as the analysis of fMRI data yet. Therefore, there are many

degrees of freedom in data preprocessing and analysis. This was the reason why this paper was published as a registered report – to decrease these possibilities a priori and not data-driven. As the GLM was preregistered, our interpretations are mainly based on the GLM results, however, we noted some additions by exploratory results and explicitly marked them as exploratory. Regarding methodological details, the main difference between GLM and amplitude analysis is that the GLM is modeling the HRF, while the amplitude analysis simply averages the signal in a certain time interval. The results of both analysis show convergent and divergent evidence.

Convergent evidence: Importantly, the amplitude results support the evidence of the GLM results for complexity effects across channels and for the interaction of age with complexity in the right MFG (channel 12). By providing convergent evidence, the exploratory analysis further supports the confirmatory analysis and validates these results. Therefore, we are sure that we can rely on these effects and discuss them as the main results of the paper. Further validations can be seen in the brain behavior-correlations, as evidence was found for an association between behavioral and neural complexity effects (see our response to the last comment below).

Divergent evidence: However, some findings differed between analyses. The amplitude analysis shows, on the one hand, evidence for main effects of age that were not captured by the GLM, and, on the other hand, less evidence for main effects of operation compared to the GLM. The reason might be that the time course of the fNIRS signal reflecting the hemodynamic response function varies, particularly for such complex cognitive tasks such as arithmetic where different time windows might capture different processing stages. This was confirmed by the exploratory analysis on peak latency, which revealed evidence for such temporal differences depending on age, operation, complexity, and brain region.

To clarify the role of the different analyses, we added the following text to the results section: "Exploratory results of amplitude and peak latency analyses are reported in S1 Supplementary Material (Tables E and F). The amplitude analyses provided convergent evidence for the GLM results, particularly for complexity effects across channels and the interaction between age group and complexity in the right MFG. However, the amplitude analysis also revealed age effects not captured by the GLM and less evidence for operation effects, possibly because the time course of the fNIRS signal and the underlying hemodynamic response varied depending on age, operation, complexity, and brain region, as further supported by the peak latency analysis." (p. 23)

3. Finally, theoretically speaking, I find the rationale behind examining arithmetic problem solving across lifespan a bit weak. Is arithmetic problem solving a good way to understand long-term memory loss in aging population? Or working memory deficiency? The older adults in fact performed as good as or even slightly better than the younger adults. If the authors are targeting at working memory, there are other tasks (e.g., n-back, Stroop) better suited for this purpose. As I mentioned in point 1, arithmetic problem solving involves many cognitive abilities. Its neural basis is not just located in IFG/MFG or IPS/SMG/AG but rather distributed across the brain. I'm not against the findings that children engaged more frontal areas and adults engaged more parietal cortex during arithmetic problem solving. However, cognitive maturation/aging includes changes in many brain systems. It's not just merely arithmetic shifted from frontal cortex to parietal cortex. I'd recommend the authors to be more careful on this theoretical point when making their hypotheses, given they are using fNIRS, which can be a quite localized method.

Reply: This study addresses arithmetic processing across the lifespan, a domain that has been mostly neglected in lifespan and aging research. A key characteristic of arithmetic, in contrast to many other cognitive domains such as working memory, is that core skills are largely preserved into older adulthood. Indeed, general cognitive functions such as processing speed typically decline with age and thus also affect the speed of arithmetic problem solving. However, even

though working memory capacity also decreases during aging and working memory is necessary for carry/borrow operations, domain-specific functions such as place-value processing appear to remain stable and may even benefit from lifelong practice and experience – resulting in smaller carry/borrow effects (Avcil & Artemenko, 2025). This combination of domain-general decline and domain-specific preservation makes arithmetic a relevant case for investigating how cognitive performance and its neural correlates are maintained or reorganized across the lifespan. In response, we extended the conclusions (p. 30): "Hence, arithmetic skills acquired in school are maintained and used throughout life, distinguishing arithmetic from many other cognitive domains that typically exhibit age-related declines."

Instead of substantial changes in the introduction at this stage, we further extended the discussion to show that this stability was found regardless of general changes in the brain during lifespan development (p. 26): "Due to the high task demands, age-related changes in brain activation – such as the gradual developmental shift from frontal to parietal activation or general developmental changes – were not observed, that might be limited to single-digit arithmetic."

Minor points:

1. The authors measured many other cognitive abilities behaviorally, including processing speed, working memory, verbal and non-verbal intelligence. Any correlations between these cognitive abilities and arithmetic performance or brain activities?

Reply: We thank the reviewer for this comment. As suggested, we calculated brain-behavior correlations between cognitive measures (processing speed, working memory, verbal/non-verbal intelligence), behavioral carry/borrow effects (ACC, RT), and neural carry/borrow effects (for all channels) for all age groups. Results can be found in Tables S7-S12 in Supplementary material. We added to the manuscript a new chapter on brain-behavior correlations (p. 23):

"In an exploratory analysis, brain-behavior correlations were calculated between cognitive measures (processing speed, working memory, verbal/non-verbal intelligence), behavioral carry/borrow effects (ACC, RT), and neural carry/borrow effects for each age group (see Supplementary Material, Tables S7-S12). Concerning behavioral effects, higher verbal intelligence was associated with larger borrow effects (ACC) in children and younger adults, higher verbal intelligence and working memory (ACC) with smaller carry or borrow effects (RT) in younger adults, and higher non-verbal intelligence and processing speed with larger carry or borrow effects (ACC) in older adults. Concerning neural effects, higher verbal intelligence and working memory (ACC) were associated with smaller borrow effects in the right IFG in children, higher working memory (ACC) with smaller carry effects in the left SMG and with smaller borrow effects in the left IFG in younger adults, higher non-verbal intelligence with larger borrow effects in the right IPS in older adults, and higher processing speed with larger borrow effects in the left MFG and right AG in older adults. Concerning the relation between behavioral (RT) and neural effects, carry effects were positively associated in the right MFG and left IPS in younger adults, borrow effects in the left IFG and MFG in younger adults, and carry effects in the bilateral MFG in older adults."
