# Supplementary material for: No developmental fronto-parietal shift in brain activation during mental arithmetic across the lifespan: A registered report
Source: PLoS One. 2026 Aug 5;21(8):e0353139. doi: 10.1371/journal.pone.0353139 (PMC13440826; doi:10.1371/journal.pone.0353139)
Supplement: Peer review history

# Peer review: Decision Letter 0

**DOI:** 10.1371/journal.pone.0353139.r001

**Authors:** Rei Akaishi

26 Apr 2026

PONE-D-26-04047

No developmental fronto-parietal shift in brain activation during mental arithmetic across the lifespan: A Registered Report

PLOS One

Dear Dr. Artemenko,

Thank you for submitting your manuscript to PLOS ONE. After careful consideration, we feel that it has merit but does not fully meet PLOS ONE’s publication criteria as it currently stands. Therefore, we invite you to submit a revised version of the manuscript that addresses the points raised during the review process.

The manuscript addresses an interesting question and the dataset has potential value, but both reviewers raise substantial concerns about the theoretical framing, the link between hypotheses and task design, the interpretation of null results, and the justification of the imaging/analysis approach. These issues preclude acceptance in the current form. However, I believe they may be addressable through a major revision that substantially narrows the claims, clarifies the rationale, and revises the interpretation in line with the actual inferential limits of the study.

Please submit your revised manuscript by Jun 10 2026 11:59PM. If you will need more time than this to complete your revisions, please reply to this message or contact the journal office at plosone@plos.org. When you're ready to submit your revision, log on to https://www.editorialmanager.com/pone/ and select the ’submissions Needing Revision' folder to locate your manuscript file.

Please include the following items when submitting your revised manuscript:

- A letter that responds to each point raised by the academic editor and reviewer(s). You should upload this letter as a separate file labeled 'Response to Reviewers'.
- A marked-up copy of your manuscript that highlights changes made to the original version. You should upload this as a separate file labeled 'Revised Manuscript with Track Changes'.
- An unmarked version of your revised paper without tracked changes. You should upload this as a separate file labeled 'Manuscript'.

If you would like to make changes to your financial disclosure, please include your updated statement in your cover letter. Guidelines for resubmitting your figure files are available below the reviewer comments at the end of this letter.

If applicable, we recommend that you deposit your laboratory protocols in protocols.io to enhance the reproducibility of your results. Protocols.io assigns your protocol its own identifier (DOI) so that it can be cited independently in the future. For instructions see: https://journals.plos.org/plosone/s/submission-guidelines#loc-laboratory-protocols. Additionally, PLOS ONE offers an option for publishing peer-reviewed Lab Protocol articles, which describe protocols hosted on protocols.io. Read more information on sharing protocols at https://plos.org/protocols?utm_medium=editorial-email&utm_source=authorletters&utm_campaign=protocols.

As the corresponding author, your ORCID iD is verified in the submission system and will appear in the published article. PLOS supports the use of ORCID, and we encourage all coauthors to register for an ORCID iD and use it as well. Please encourage your coauthors to verify their ORCID iD within the submission system before final acceptance, as unverified ORCID iDs will not appear in the published article. *Only*  the individual author can complete the verification step; PLOS staff *cannot*  verify ORCID iDs on behalf of authors.

We look forward to receiving your revised manuscript.

Kind regards,

Rei Akaishi

Academic Editor

PLOS One

Journal Requirements:

When submitting your revision, we need you to address these additional requirements.

1. Please ensure that your manuscript meets PLOS ONE’s style requirements, including those for file naming. The PLOS ONE style templates can be found at

https://journals.plos.org/plosone/s/file?id=wjVg/PLOSOne_formatting_sample_main_body.pdf and

https://journals.plos.org/plosone/s/file?id=ba62/PLOSOne_formatting_sample_title_authors_affiliations.pdf

2. Thank you for stating the following financial disclosure: [This research project was funded by the German Research Foundation (DFG, grant number: 468460838, AR 1500/1-1) and by the Institutional Strategy of the University of Tuebingen (Deutsche Forschungsgemeinschaft, ZUK 63). CA was further supported by the European Social Fund and the Ministry of Science, Research and the Arts Baden-Wuerttemberg, by the German Research Foundation (DFG, grant number: 513458453, AR 1500/2-1), by the LEAD Graduate School & Research Network (GSC1028, funded within the Excellence Initiative of the German federal and state governments), and by the Tuebingen Postdoc Academy for Research on Education (PACE) at the Hector Research Institute of Education Sciences and Psychology, Tuebingen (PACE is funded by the Baden-Wuerttemberg Ministry of Science, Research and the Arts).].

Please state what role the funders took in the study.  If the funders had no role, please state: ""The funders had no role in study design, data collection and analysis, decision to publish, or preparation of the manuscript.""

If this statement is not correct you must amend it as needed.

Please include this amended Role of Funder statement in your cover letter; we will change the online submission form on your behalf.

3. Please include your full ethics statement in the ‘Methods’ section of your manuscript file. In your statement, please include the full name of the IRB or ethics committee who approved or waived your study, as well as whether or not you obtained informed written or verbal consent. If consent was waived for your study, please include this information in your statement as well.

4. Please include captions for your Supporting Information files at the end of your manuscript, and update any in-text citations to match accordingly. Please see our Supporting Information guidelines for more information: http://journals.plos.org/plosone/s/supporting-information.

5. If the reviewer comments include a recommendation to cite specific previously published works, please review and evaluate these publications to determine whether they are relevant and should be cited. There is no requirement to cite these works unless the editor has indicated otherwise.

Additional Editor Comments:

The manuscript addresses an interesting question and the dataset has potential value, but both reviewers raise substantial concerns about the theoretical framing, the link between hypotheses and task design, the interpretation of null results, and the justification of the imaging/analysis approach. These issues preclude acceptance in the current form. However, I believe they may be addressable through a major revision that substantially narrows the claims, clarifies the rationale, and revises the interpretation in line with the actual inferential limits of the study.

[Note: HTML markup is below. Please do not edit.]

Reviewers' comments:

Reviewer’s Responses to Questions

**Comments to the Author**

1. Does the manuscript adhere to the experimental procedures and analyses described in the Registered Report Protocol?

If the manuscript reports any deviations from the planned experimental procedures and analyses, those must be reasonable and adequately justified.

Reviewer #1: Yes

Reviewer #2: Yes

**********

2. If the manuscript reports exploratory analyses or experimental procedures not outlined in the original Registered Report Protocol, are these reasonable, justified and methodologically sound?

A Registered Report may include valid exploratory analyses not previously outlined in the Registered Report Protocol, as long as they are described as such.

Reviewer #1: Yes

Reviewer #2: Partly

**********

3. Are the conclusions supported by the data and do they address the research question presented in the Registered Report Protocol?

The manuscript must describe a technically sound piece of scientific research with data that supports the conclusions. The conclusions must be drawn appropriately based on the research question(s) outlined in the Registered Report Protocol and on the data presented.

Reviewer #1: Yes

Reviewer #2: Partly

**********

4. Have the authors made all data underlying the findings in their manuscript fully available?

The PLOS Data policy requires authors to make all data underlying the findings described in their manuscript fully available without restriction, with rare exception (please refer to the Data Availability Statement in the manuscript PDF file). The data should be provided as part of the manuscript or its supporting information, or deposited to a public repository. For example, in addition to summary statistics, the data points behind means, medians and variance measures should be available. If there are restrictions on publicly sharing data—e.g. participant privacy or use of data from a third party—those must be specified.

Reviewer #1: Yes

Reviewer #2: Yes

**********

5. Is the manuscript presented in an intelligible fashion and written in standard English?

*PLOS ONE*  does not copyedit accepted manuscripts, so the language in submitted articles must be clear, correct, and unambiguous. Any typographical or grammatical errors should be corrected at revision, so please note any specific errors here.

Reviewer #1: Yes

Reviewer #2: Yes

**********

6. Review Comments to the Author

Please use the space provided to explain your answers to the questions above. (Please upload your review as an attachment if it exceeds 20,000 characters)

Reviewer #1: This is an interesting and overall well-written manuscript. The study examines arithmetic processing across the lifespan by comparing elementary school children, younger adults, and older adults, with the goal of characterizing behavioral and neural correlates of arithmetic at different stages: during initial learning, after consolidation, and later in aging. The focus on two-digit addition and subtraction problems with and without carrying/borrowing is potentially valuable. At the same time, several aspects of the theoretical framing, hypothesis formulation, and interpretation require clarification and greater precision. I encourage the authors to strengthen the conceptual rationale for the study design, more clearly justify how the chosen task can test the proposed hypotheses, and moderate some of the claims in the discussion.

Major comments

1. The rationale for the study should not rely on novelty alone

On p. 5, the manuscript states that because neuroimaging research on arithmetic in older adults has not been conducted before, the project opens a new research field in numerical cognition. I do not find this argument sufficiently convincing on its own. The importance of the study should be grounded primarily in its theoretical contribution and in the extent to which the design can adjudicate between competing predictions. I recommend revising this section to explain more clearly why studying this shift including older adults is relevant, rather than emphasizing that the topic has not previously been studied.

2. The framing of single-digit versus two-digit arithmetic needs to be reconsidered

The paragraph on p. 5 about the limitations of prior work focusing on single-digit arithmetic is currently not fully convincing. I would avoid characterizing that literature as “unfortunate.” In fact, single-digit arithmetic is highly relevant in elementary school and has been central to prior developmental work on arithmetic-related brain activation. More importantly, the manuscript should engage more directly with the implications of using two-digit addition and subtraction rather than single-digit arithmetic. This is especially critical because the fronto-parietal shift literature invoked by the authors appears to derive largely from studies using single-digit operations (e.g. Rivera et al., 2005). Two-digit arithmetic may recruit additional procedural, working-memory, and place-value-related processes, which may reduce comparability with that earlier literature. As a result, it is not yet clear whether the present design is ideally suited to test predictions derived from prior evidence on the developmental fronto-parietal shift.

I therefore recommend that the authors revise this section to justify more explicitly the use of two-digit arithmetic for the present research questions, acknowledge more clearly how this choice differs from the literature on which the hypotheses are based, and discuss whether the use of two-digit operations may itself contribute to the absence of evidence for the expected shift.

3. The concept of a fronto-parietal shift “continuing during aging” needs clarification

The manuscript repeatedly refers to whether the developmental fronto-parietal shift “continues during aging.” I found this formulation conceptually unclear. A shift typically refers to a change from one state or pattern to another. Describing such a shift as “continuing” during aging is difficult to interpret unless the authors mean that the shift is gradual and extends across additional stages of the lifespan. The authors should define more precisely what they mean by this idea. For example, do they predict a continued decrease in frontal reliance and increase in parietal reliance beyond young adulthood? A maintenance of the adult pattern into older age? This issue should be clarified both in the Introduction and in the Discussion.

4. The link between carrying/borrowing and the fronto-parietal shift requires a stronger rationale

On p. 7, the manuscript suggests that carry and borrow effects are associated with increased frontal and parietal activation, and appears to use this as part of the rationale for including these manipulations. However, it is not yet clear why these manipulations are necessary for testing hypotheses about the fronto-parietal shift. The authors should explain more explicitly: 1) why carry and borrow were included. 2) whether these manipulations are central to the main hypotheses or secondary to them. 3) how complexity-related effects are expected to inform the broader question of age-related changes in arithmetic processing. In the current version of the manuscript, the connection between arithmetic complexity effects and the proposed lifespan fronto-parietal account remains underdeveloped.

5. Some hypotheses are difficult to interpret and need to be reformulated

Several hypotheses would benefit from greater precision.

On p. 8, the hypothesis that children should show increased frontal activation and less parietal activation than younger adults is presented as following from previous work on the developmental fronto-parietal shift. However, because that literature is based primarily on single-digit arithmetic, the authors should acknowledge that applying the same prediction to two-digit arithmetic requires an additional assumption.

Also on p. 8, the paragraph discussing older adults is difficult to follow because it combines distinct possibilities into a single sentence. I suggest separating the alternatives more clearly. For instance, one possibility is that arithmetic processing is relatively preserved in aging, which would predict a pattern similar to that of younger adults. A different possibility is that older adults rely more strongly on compensatory mechanisms, which may predict greater frontal recruitment. These alternatives should be presented distinctly.

Likewise, the section on the predicted developmental trajectory of carry and borrow effects is currently confusing. The manuscript proposes alternative patterns, but it is not sufficiently clear what neural changes are expected in each case, or how the data would distinguish between them. This should be rewritten more explicitly.

6. The discussion should interpret the null evidence more cautiously

My main concern in the Discussion is that several conclusions are stated too strongly relative to the design and method. The authors do note that the use of fNIRS may have limited the ability to detect a fronto-parietal shift, which is appropriate. However, I think the discussion should also acknowledge additional reasons why such evidence may not have been observed in the present study. In particular, the authors should discuss more fully the use of two-digit rather than single-digit arithmetic. Because two-digit addition and subtraction can recruit a mixture of strategies and impose broader cognitive demands, this paradigm may not be optimal for isolating a developmental shift toward parietal specialization. Relatedly, if the task maintains procedural demands at a relatively high level for all groups, age differences in frontal recruitment may be attenuated or masked. I suggest that authors are more clear about the possibility that task complexity may have sustained frontal control demands across all age groups, thereby making developmental differences harder to detect.

For these reasons, statements such as “our results challenge the existence of the fronto-parietal shift” seem too strong. A more appropriate conclusion would be something along the following lines: “We did not observe evidence for the fronto-parietal shift in this sample using this fNIRS paradigm with two-digit addition and subtraction.” That wording is more consistent with the actual design and with the limits of the inferences that can be drawn.

7. Some claims about IPS activation are overstated

On p. 26, the manuscript states that the stability of IPS activation during aging points to functional specialization for number magnitude processing that is maintained throughout adulthood without deterioration. I think this statement goes beyond what the present data can support. Reliance on IPS during addition and subtraction does not, by itself, permit strong conclusions about number magnitude refinement. I recommend revising this passage more cautiously and tying the interpretation more directly to the specific task demands used here.

Minor comments

• p. 4, line 84: I suggest revising the sentence to:

“Moreover, the question arises as to how these developmental changes progress during aging.”

• p. 5, line 94: This paragraph would benefit from rewriting for clarity and balance. In particular, I would avoid implying that the focus on single-digit arithmetic in prior work is inherently problematic.

• p. 8, line 195: Please provide a clearer rationale for the prediction that carry and borrow effects should be categorical in children, mixed in younger adults, and continuous in older adults.

• p. 22, Table 2: Please define all abbreviations in the table note, including “ch.” Also, centering the contents may improve readability.

• p. 24, Figures 3 and 4: These figures are currently very difficult to read. I recommend separating frontal and posterior results into different panels or figures and making significant effects easier to identify visually.

Overall, I think this manuscript addresses an interesting question and has the potential to make a valuable contribution. To strengthen the paper, I encourage the authors to sharpen the theoretical rationale, clarify the hypotheses, and adopt a more cautious interpretation of the findings. In particular, the manuscript would benefit from a more explicit discussion of how the use of two-digit arithmetic and fNIRS affects comparability with prior literature on the developmental fronto-parietal shift.

Reviewer #2: The manuscript examines addition and subtraction problem solving in elementary school students, younger adults, and older adults using fNIRS. The authors hypothesized a fronto-parietal shift during arithmetic problem solving across age and the complexity (carrying/borrowing) of the problems should result in enhanced activity in frontal areas, especially in children. The main findings did not support the hypotheses: no fronto-parietal shift was found across age groups and the level of enhancement due to complexity in the frontal area was smaller in children than in adults in right MFG. Overall, I find myself confused by the results and not fully convinced by the authors’ interpretation. The theoretical setup of the manuscript is also a bit unclear. I will explain my points in detail below.

Major concerns:

1. Methodologically I’m concerned whether fNIRS is the best tool to investigate this research question and whether the authors picked the right target regions in the frontal cortex. Arithmetic problem solving involves distributed brain regions, mainly including ventral visual pathway, language system, medial temporal memory system, parietal cortex, and frontal cognitive control system. Only examining the lateral frontal cortex and parietal cortex could result in findings hard to interpret. Moreover, the authors highlighted the relationship between frontal cortex and working memory. I’m worried that the authors might have missed the important dorsomedial prefrontal cortex/dorsal ACC and insula (salience network) as these regions are well known for their domain general functions and have been frequently found during arithmetic problem solving (see Sokolowski, Hawes, & Ansari, 2022 and Unal, Park, Simsek, Menon, & Geary, 2025 meta-analysis).

2. I’m having a hard time understanding the rationale for the exploratory analysis on the mean amplitude and peak latency. I’m not an expert on fNIRS but more on fMRI. Conceptually this exploratory analysis is analyzing the amplitude and peak latency of blood oxygen level change. Indeed, the exploratory findings support the hypotheses better than the GLM findings. However, many of the exploratory results contradict with the GLM results. What does that mean? Which analysis should we trust and why? The authors need to provide stronger rationale for the exploratory analysis.

3. Finally, theoretically speaking, I find the rationale behind examining arithmetic problem solving across lifespan a bit weak. Is arithmetic problem solving a good way to understand long-term memory loss in aging population? Or working memory deficiency? The older adults in fact performed as good as or even slightly better than the younger adults. If the authors are targeting at working memory, there are other tasks (e.g., n-back, Stroop) better suited for this purpose. As I mentioned in point 1, arithmetic problem solving involves many cognitive abilities. Its neural basis is not just located in IFG/MFG or IPS/SMG/AG but rather distributed across the brain. I’m not against the findings that children engaged more frontal areas and adults engaged more parietal cortex during arithmetic problem solving. However, cognitive maturation/aging includes changes in many brain systems. It’s not just merely arithmetic shifted from frontal cortex to parietal cortex. I’d recommend the authors to be more careful on this theoretical point when making their hypotheses, given they are using fNIRS, which can be a quite localized method.

Minor points:

1. The authors measured many other cognitive abilities behaviorally, including processing speed, working memory, verbal and non-verbal intelligence. Any correlations between these cognitive abilities and arithmetic performance or brain activities?

**********

7. PLOS authors have the option to publish the peer review history of their article (what does this mean?). If published, this will include your full peer review and any attached files.

If you choose “no”, your identity will remain anonymous but your review may still be made public.

**Do you want your identity to be public for this peer review?**  For information about this choice, including consent withdrawal, please see our Privacy Policy.

Reviewer #1: No

Reviewer #2: No

**********

[NOTE: If reviewer comments were submitted as an attachment file, they will be attached to this email and accessible via the submission site. Please log into your account, locate the manuscript record, and check for the action link "View Attachments". If this link does not appear, there are no attachment files.]

To ensure your figures meet our technical requirements, please review our figure guidelines: https://journals.plos.org/plosone/s/figures

You may also use PLOS’s free figure tool, NAAS, to help you prepare publication quality figures: https://journals.plos.org/plosone/s/figures#loc-tools-for-figure-preparation.

NAAS will assess whether your figures meet our technical requirements by comparing each figure against our figure specifications.

# Author comment: Author response to Decision Letter 1

**DOI:** 10.1371/journal.pone.0353139.r002

29 May 2026

see comments in the response letter

Attachment Submitted filename: life-neuro_response-letter_rev1_3.pdf

# Peer review: Decision Letter 1

**DOI:** 10.1371/journal.pone.0353139.r003

**Authors:** Rei Akaishi, Rei Akaishi

19 Jun 2026

No developmental fronto-parietal shift in brain activation during mental arithmetic across the lifespan: A Registered Report

PONE-D-26-04047R1

Dear Dr. Artemenko,

We’re pleased to inform you that your manuscript has been judged scientifically suitable for publication and will be formally accepted for publication once it meets all outstanding technical requirements.

Within one week, you’ll receive an e-mail detailing the required amendments. When these have been addressed, you’ll receive a formal acceptance letter and your manuscript will be scheduled for publication.

An invoice will be generated when your article is formally accepted. Please note, if your institution has a publishing partnership with PLOS and your article meets the relevant criteria, all or part of your publication costs will be covered. Please make sure your user information is up-to-date by logging into Editorial Manager at Editorial Manager® and clicking the ‘Update My Information' link at the top of the page. For questions related to billing, please contact billing support.

If your institution or institutions have a press office, please notify them about your upcoming paper to help maximize its impact. If they’ll be preparing press materials, please inform our press team as soon as possible -- no later than 48 hours after receiving the formal acceptance. Your manuscript will remain under strict press embargo until 2 pm Eastern Time on the date of publication. For more information, please contact onepress@plos.org.

Kind regards,

Rei Akaishi

Academic Editor

PLOS One

Additional Editor Comments (optional):

Reviewers' comments:

Reviewer’s Responses to Questions

**Comments to the Author**

1. Does the manuscript adhere to the experimental procedures and analyses described in the Registered Report Protocol?

If the manuscript reports any deviations from the planned experimental procedures and analyses, those must be reasonable and adequately justified.

Reviewer #2: Yes

**********

2. If the manuscript reports exploratory analyses or experimental procedures not outlined in the original Registered Report Protocol, are these reasonable, justified and methodologically sound?

A Registered Report may include valid exploratory analyses not previously outlined in the Registered Report Protocol, as long as they are described as such.

Reviewer #2: Yes

**********

3. Are the conclusions supported by the data and do they address the research question presented in the Registered Report Protocol?

The manuscript must describe a technically sound piece of scientific research with data that supports the conclusions. The conclusions must be drawn appropriately based on the research question(s) outlined in the Registered Report Protocol and on the data presented.

Reviewer #2: Yes

**********

4. Have the authors made all data underlying the findings in their manuscript fully available?

The PLOS Data policy requires authors to make all data underlying the findings described in their manuscript fully available without restriction, with rare exception (please refer to the Data Availability Statement in the manuscript PDF file). The data should be provided as part of the manuscript or its supporting information, or deposited to a public repository. For example, in addition to summary statistics, the data points behind means, medians and variance measures should be available. If there are restrictions on publicly sharing data—e.g. participant privacy or use of data from a third party—those must be specified.

Reviewer #2: Yes

**********

5. Is the manuscript presented in an intelligible fashion and written in standard English?

*PLOS ONE*  does not copyedit accepted manuscripts, so the language in submitted articles must be clear, correct, and unambiguous. Any typographical or grammatical errors should be corrected at revision, so please note any specific errors here.

Reviewer #2: Yes

**********

6. Review Comments to the Author

Please use the space provided to explain your answers to the questions above. (Please upload your review as an attachment if it exceeds 20,000 characters)

Reviewer #2: I appreciate the authors' effort on addressing my comments and provide additional analysis. All my concerns are addressed.

**********

7. PLOS authors have the option to publish the peer review history of their article (what does this mean?). If published, this will include your full peer review and any attached files.

If you choose “no”, your identity will remain anonymous but your review may still be made public.

**Do you want your identity to be public for this peer review?**  For information about this choice, including consent withdrawal, please see our Privacy Policy.

Reviewer #2: No

**********

# Editor report: Acceptance letter

**DOI:** 10.1371/journal.pone.0353139.r004

**Authors:** Rei Akaishi, Rei Akaishi

PONE-D-26-04047R1

PLOS One

Dear Dr. Artemenko,

I'm pleased to inform you that your manuscript has been deemed suitable for publication in PLOS One. Congratulations! Your manuscript is now being handed over to our production team.

At this stage, our production department will prepare your paper for publication. This includes ensuring the following:

* All references, tables, and figures are properly cited

* All relevant supporting information is included in the manuscript submission,

* There are no issues that prevent the paper from being properly typeset

You will receive further instructions from the production team, including instructions on how to review your proof when it is ready. Please keep in mind that we are working through a large volume of accepted articles, so please give us a few days to review your paper and let you know the next and final steps.

Lastly, if your institution or institutions have a press office, please let them know about your upcoming paper now to help maximize its impact. If they'll be preparing press materials, please inform our press team within the next 48 hours. Your manuscript will remain under strict press embargo until 2 pm Eastern Time on the date of publication. For more information, please contact onepress@plos.org.

You will receive an invoice from PLOS for your publication fee after your manuscript has reached the completed accept phase. If you receive an email requesting payment before acceptance or for any other service, this may be a phishing scheme. Learn how to identify phishing emails and protect your accounts at https://explore.plos.org/phishing.

If we can help with anything else, please email us at customercare@plos.org.

Thank you for submitting your work to PLOS ONE and supporting open access.

Kind regards,

PLOS ONE Editorial Office Staff

on behalf of

Dr. Rei Akaishi

Academic Editor

PLOS One
